# The Extracellular Matrix and Biocompatible Materials in Glioblastoma Treatment

**DOI:** 10.3389/fbioe.2019.00341

**Published:** 2019-11-19

**Authors:** Andrei Belousov, Sergei Titov, Nikita Shved, Mikhail Garbuz, Grigorii Malykin, Valeriia Gulaia, Alexander Kagansky, Vadim Kumeiko

**Affiliations:** ^1^School of Biomedicine, Far Eastern Federal University, Vladivostok, Russia; ^2^School of Natural Sciences, Far Eastern Federal University, Vladivostok, Russia; ^3^A.V. Zhirmunsky National Scientific Center of Marine Biology, Far Eastern Branch of Russian Academy of Sciences, Vladivostok, Russia

**Keywords:** glioblastoma, biocompatible material, extracellular matrix, cancer treatment, drug delivery, CNS reconstruction, neuroregeneration

## Abstract

During cancer genesis, the extracellular matrix (ECM) in the human brain undergoes important transformations, starting to resemble embryonic brain cell milieu with a much denser structure. However, the stiffness of the tumor ECM does not preclude cancer cells from migration. The importance of the ECM role in normal brain tissue as well as in tumor homeostasis has engaged much effort in trials to implement ECM as a target and an instrument in the treatment of brain cancers. This review provides a detailed analysis of both experimental and applied approaches in combined therapy for gliomas in adults. In general, matrix materials for glioma treatment should have properties facilitating the simplest delivery into the body. Hence, to deliver an artificial implant directly into the operation cavity it should be packed into a gel form, while for bloodstream injections matrix needs to be in the form of polymer micelles, nanoparticles, etc. Furthermore, the delivered material should mimic biomechanical properties of the native tissue, support vital functions, and slow down or stop the proliferation of surrounding cells for a prolonged period. The authors propose a two-step approach aimed, on the one hand, at elimination of remaining cancer cells and on the other hand, at restoring normal brain tissue. Thereby, the first bioartificial matrix to be applied should have relatively low elastic modulus should be loaded with anticancer drugs, while the second material with a higher elastic modulus for neurite outgrowth support should contain specific factors stimulating neuroregeneration.

## Introduction

Malignant neoplasms are among the most important global health problems. There are many different types of malignant neoplasms, which are divided depending on the location of the tumor, the stage of development and other characteristics. However, among the most dangerous group of tumors are malignant neoplasms of the brain due to their significant impact on the patient's life in physical, psychological and neurological aspects (Ng et al., 2019), which can lead to permanent disability (Kunert et al., 2011).

Among all brain tumors, glioblastoma multiforme (GBM) is the most common one in adults as well as the most aggressive. GBM has a poor prognosis, since the tumor is considered incurable and the median patient survival is 15 months. In addition, there are no preventive measures that could preclude the development of the disease or improve its outcome (Bastiancich et al., 2018; Pinel et al., 2019). Pediatric gliomas (0–19 years old) deserve special attention, although their incidence is relatively low, around 3% of all brain and CNS tumors (Ostrom et al., 2014). The development of pediatric gliomas has several hallmark differences, for example, they carry distinct marker mutation histone H3 K27M substitution and more frequently exhibit p53 alterations, while PTEN deletion and EGFR amplification are more rare compared to primary adult GBM (Suri et al., 2009), however more detailed studies on the differences between pediatric and adult gliomas have already been cited in review articles (Fangusaro, 2012; Sturm et al., 2017). Current manuscript focuses only on the treatment of adult gliomas.

Standard treatment of a malignant neoplasm of the brain involves visualization of the tumor, its surgical resection without causing neurological damage, and is accompanied by therapeutic treatment (Yamahara et al., 2010).

After the diagnosis of glioblastoma and its surgical removal, the patient undergoes subsequent treatment with radiotherapy and concomitant chemotherapy with various cytotoxic drugs for around 6 weeks. However, during the development of a tumor its cells aggressively migrate and grow into surrounding structures of the brain, which makes it impossible to completely remove the internal neoplasm surgically leading to almost 100% relapse rate. In addition, long-term post-operative recovery may delay the subsequent stages of treatment, which contributes to the proliferation of residual tumor cells, leading to recurrence of GBM within 2 years after the initial diagnosis in most patients (Bastiancich et al., 2016b; Pinel et al., 2019).

Summarizing all the facts mentioned above, the traditional approach to treating cancer focuses only on tumor cells, ignoring their non-cellular environment, specifically, their extracellular matrix (ECM). However, it has been proven that the key role in the development and progression of a tumor is not played by tumor cells themselves, but by the ECM and the tumor stem cell niche it forms (Kim et al., 2005; Mikhailova et al., 2018). Understanding this role is crucial for predicting the fate and behavior of the remaining cancer cells and the dynamics of recovery (Mercier, 2016; Reinhard et al., 2016).

ECM undergoes changes as long as life endures. Specific molecular architecture of ECM provides for the differentiation and migration of neural progenitor cells in early development. Upon transition to adulthood, a partial change in the molecular pattern and matrix composition occurs, which supports lower migration ability, low proliferative activity, but retains axon guidance. During cancer genesis, the adhesive properties of the matrix are weakened and the matrix is remodeled, which leads to its juvenile composition, but its rigidity increases significantly, both in comparison with the juvenile and adult state. The use of biocompatible matrix materials, both natural and synthetic, on the one hand creates a new microenvironment in cells, becoming able to deliver anticancer therapeutic agents (drugs, cells, genetic engineering constructs), and, on the other hand, due to the “cell-matrix” interaction, can contribute to the remodeling of native ECM in the lesion, ensuring the progress of a “healthy” microenvironment, and the resumption of control of cell proliferation. The described transformations of the natural matrix and the necessary changes in the composition of biocompatible matrix materials during the treatment of gliomas are presented in this paper.

The use of various synthetic or natural materials that mimic normal ECM and create a microenvironment for cells in the resection cavity after removing a brain tumor can significantly improve the prognosis, which leads to a great interest in research in this area, and various types of matrix materials and methods are being developed.

In this article, we overview (i) the structure of normal ECM, which is a model for artificial matrix materials to imitate (ii) changes of ECM molecular profile during carcinogenesis to better understand the effect of tumors on the extracellular space, (iii) the possibility of influencing the tumor itself through various matrices by its molecular environment, and (iv) the latest trends in choosing matrix materials for creating implants.

## The Extracellular Matrix of the Human Brain

Human brain is a very complex structure, in comparison to other organs, in terms of its tissue architecture at the first place. Brain neural tissue functions as a dynamic network—beneficial synaptic connections need to be maintained, and other reconstructed to match changing input stimuli.

Cell–cell interactions in the brain, similarly to other tissues, are based on direct contacts via cadherins and signaling receptors, as well as cell–matrix interactions with the ECM (Senkov et al., 2014). Various molecules compose the neural ECM in the brain (Table 1).

**Table 1:** The key components of the brain ECM (from Novak and Kaye, 2000, modified).

**Component**	**Size of core protein subunit in kDa**	**Glycosylation**	**Gene location**	**Onthology/Functions**
**Proteoglycans**				
Aggrecan (ACAN)	210	CS + KS	15q21–q26	PNNs in adult brain; member of the neural stem cell niche; gliogenesis
Agrin	225	HS	1p36.33	Basal lamina in microvessels; component of BBB
Biglycan	42	CS/DS	Xq27–ter	Gliogenesis, collagen matrix assembly
Brevican	145	HS	1q23.1	PNNs in adult brain
Decorin	40	CS/DS	12q21–q22	Assembly of collagen components of the ECM
Glypican family (membrane-anchored)	60–70	HS	Multiple locations	Neuronal development, interaction with laminin
Lumican	40	N	12q21.33	Organizer of collagen fibrils in the ECM; can inhibit MMPs
Neurocan	136	CS/DS, N, O	19	PNNs in young brain
Perlecan	400	HS, N	1p36.12	Basal lamia component, important component of the stem cell niche; gliogenesis
Phosphacan (DSD-1-PG) (membrane-anchored)	380/170	CS, N	7q31.32	Neural development, plasticity, regeneration
Syndecan family, including Syndecan-2 (SDC2) (membrane-anchored)	42	HS, O	8q22.1	Cell proliferation, cell migration and cell-matrix interactions. Typical to brain cancer stem cells, but not to NSCs
Versican	265	CS/DS, N	5q14.3	PNNs in young brain
Hyaluronan (Non-proteoglycan GAG)	Not applicable	Not applicable	Not applicable	Tissue hydration, cell migration routes
**Glycoproteins**				
Link protein	43–49	N	5q14.3	PNNs
Reelin	388	N	7q22.1	Neuronal migration, axonal and dendrite growth, synaptic plasticity
Tenascin-C	240	N	9q32–q34	PNNs in young brain
Tenascin-R	150	N	1q22–q24	PNNs in adult brain
Tenascin-X	500	N	6p21.3	PNNs in young brain
**Fibrous glycoproteins**				
Collagens, 28 types (different subunits forming homo- and heterotrimers)	60–340 (most types 140)	N	Several locations encode subunits	Core component of the ECM in all tissues
Esp., Collagen type IV	Complex of 3 chains, 180 kDa per chain	N	13q34, 2q36.3, Xq22.3 (2 isoforms per location)	Component of the basal lamina, interconnecting hyaluronan strands
Fibronectin	274	N	2p14–p16	Component of the basal lamina
Laminin in several trimeric forms	500–800	N	Several locations encode subunits	Component of the basal lamina

*BBB, blood brain barrier; CS, chondroitin sulfate; CSC, cancer stem cell; DS, dermatan sulfate; HS, heparan sulfate; KS, keratan sulfate; MMP, matrix metalloproteinases; N, N-linked glycosylation; NSC, neural stem cell; PNN, perineuronal net; O, O-linked glycosylation; ND, not determined*.

While the role of neural elements in functioning of the brain is universally recognized and extensively studied, the importance of neural ECM is less widely reviewed.

**Figure 1:**
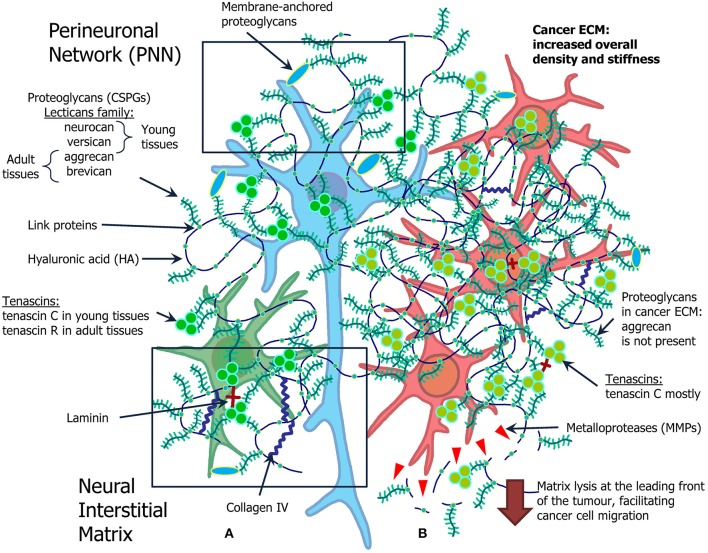
Overview of the ECM in the normal brain and in brain tumors. **(A)** Normal ECM profile in the adult CNS (left): normal neuron (blue) and glial cell (green) are surrounded by proteoglycans (neurocan, versican, aggrecan, brevican), hyaluronic acid, tenascins (tenascin C and tenascin R), laminin, and collagen IV. **(B)** Cancer microenvironment formed by the atypical ECM, its role in cancer stem cell niche organization and cell migration (right). Glioma cells (red) grow in ECM with increased density and stiffness, as a lot of components of normal ECM are overexpressed in glioma milieu. The most pronounced difference is expression of MMPs, selection for tenascin C mostly common for young tissue, and absence of aggrecan.

The ECM consists of hundreds of different molecules that interact in complex and highly organized ways (Figure 1A). The major classes of macromolecules in the ECM are the structural glycoproteins (such as collagens, elastins, fibronectins, and laminins), proteoglycans (e.g., heparan sulfate), and glycosaminoglycans (GAGs), such as hyaluronan. Components of the ECM interact with each other; and interactions between the matrix and cells are of a vital importance to the functioning of the tissue. Previously it was considered that the ECM plays mostly structural role in tissues, but lately it has become clear that ECM is involved in determining cell fate, cell migration, cell maturation and differentiation, cell survival, tissue homeostasis, and tumor cell invasion. Specific surface receptors are expressed by cells, which mediate these responses (Plopper, 2015). Sometimes it can be hard to determine the precise role of different matrix components, as mutant forms of these components cause early embryonic death in experimental animals (Novak and Kaye, 2000).

Different tissues have unique and specialized ECM composition and structure, which enables ECM to carry interacting with these extracellular matrix components, including receptors which are not presented in tissue-specific roles, including structural support, macromolecular filtration, cell migration, and other functions (Hay, 1993; Mecham, 2012). Brain ECM contains relatively small amounts of fibrous proteins like collagens or fibronectin but high amounts of glycosaminoglycans, either bound to proteins, thus forming proteoglycans, or unbound in the form of hyaluronan (Novak and Kaye, 2000). Although, collagens, especially collagen IV, serve as an important organizing molecule in both young and adult brain ECM. Withal, there is a family of so-called small leucine-rich proteoglycans, which includes several members, such as decorin and biglycan (family I) and lumican (family II), which take a crucial role in collagen fibrils assembly (Yu et al., 2017).

One of the very basic components of brain ECM, both adult and young, is hyaluronan, or hyaluronic acid (HA). It is a type of a non-proteoglycan GAG found in all human tissues and body fluids, and is especially abundant in embryonic tissues. HA is also synthesized during tissue regeneration in wounds, as well as during pathological processes in which cell migration plays a major role, such as cancer genesis, and especially tumor cell invasion (Bouterfa et al., 1997). Also, it is shown that concentration of HA is higher in brain tumors, such as gliomas, than in normal brain tissues (Delpech et al., 1993), this aspect will be discussed further.

Although, many key components of the adult brain ECM are also widely present in various other tissue types, brain ECM is especially GAG-rich, its structure is somewhat more amorphous (e.g., compared to that of connective tissues and basal laminae of epithelia), and different components of ECM tend to form a unique type of basic structure, a perineuronal net (PNN) (Mouw et al., 2014).

This core assembly of the adult brain extracellular matrix, which is found mainly in pericellular spaces of neurons, is believed to be the hyaluronan-lectican-tenascin-R complex. Aggrecan, brevican, neurocan, versican are chondroitin sulfate proteoglycans (CSPGs), members of lecticans family. Core proteins of these proteoglycans include a hyaluronan-binding domain and a C-type lectin domains, through which lecticans interact with carbohydrate and protein ligands in the extracellular matrix and act as linkers of these ECM molecules. In the adult brain, lecticans are thought to interact with hyaluronan and tenascin-R to form a ternary complex of PNNs (Yamaguchi, 2000). Besides these roles typical to ECM, and a probable involvement in preserving neuronal health, PNNs are thought to be critical for maintaining synaptic plasticity (Lau et al., 2013).

## The Extracellular Matrix of Brain Tumors, Changes in the Composition of the Matrix During Oncogenesis

Brain tumors, especially gliomas, trigger significant alterations of the ECM in brain tissues (Figure 1B).

Such changes include altered synthesis of the components by the tumor cells, extensive degradation of the ECM at the invasive front of the tumor, as well as an elevated level of synthesis of ECM components by normal tissues in the vicinity of the invading tumor.

Normal adult brain cells tend to display significant changes in the levels of ECM components produced in the presence of tumor cells. It was shown that normal cells have the ability to produce extracellular matrix components, such as laminin, collagen type IV and fibronectin, when confronted with invading glioma cells. At the same time, glioma cells express specific integrins and other receptors interacting with these extracellular matrix components, including receptors which are not presented in the corresponding normal cells, thus facilitating tumor cell migration and invasion (Knott et al., 1998). The relative volume of the ECM in comparison to that of cells (also referred to as the ECS volume fraction) also tends to increase, e.g., reaching 48% in grade III and IV Astrocytomas (Zamecnik, 2005); while in the normal brain this parameter is usually about 20% (Nicholson and Syková, 1998). In this regard tumors start to resemble the young brain, where the ECS volume fraction is ~40%. Such non-confined conditions are believed to contribute to tumor cells ability to migrate (Zamecnik, 2005).

Glioma cells have the ability to actively migrate using blood vessels or axons as guide paths due to interaction with the ECM (Cuddapah et al., 2014). Additionally, glioma cells can secrete their own ECM components, including HA, brevikan, tenascin-C and thrombospondin, as well as fibronectin, which are actively expressed in the ECM of the developing nervous system along cell migration paths (Bilozur and Hay, 1988). Fibronectin and HA increase the mobility of glioma cells and increase their invasiveness. Cells of high-grade gliomas synthesize hyaluronan in levels up to 20 times higher than in normal adult brain, which is comparable to or above those in fetal brain (Delpech et al., 1993). HA in tumors is also involved in cancer cells migration, as its production by tumor cells enables CD44 expressing glioma cells to migrate on hyaluronan-containing matrix, and the higher the density of CD44 receptors on cells' surface. CD44, the main cell surface receptor for HA, is thought to play an important role in altering GBM invasion through direct CD44-HA interaction (Koochekpour et al., 1995). The other hyaluronan binding proteins RHAMM and BEHAB are also expressed mostly in glioma cells, and further contribute to the invasive abilities of these tumors (Turley et al., 1994; Seidenbecher et al., 1995). Moreover, it was demonstrated that high grade gliomas abnormally express various hyaluronidases PH-20, Hyal2 (Liu et al., 1996; Novak et al., 1999), which is not observed in corresponding normal tissues. This is especially important for the tumor front edge, where lysis of different ECM components by enzymes secreted by cancer cells is critical for their migration and tumor invasion.

Furthermore CD44 is involved in formation of tumor leading front by binding the matrix metalloproteinase (MMP) MMP9. This interaction has been demonstrated to cause degradation of the collagen IV, while cells expressing both CD44 and MMP9 can form invasive tumors (Yu and Stamenkovic, 1999). Other matrix proteinases can be found in brain tumor cells, such as urokinase-type plasminogen activator (Bellail et al., 2004). Glioblastoma multiforme cells (U251) also actively produce extracellular matrix metalloproteinase inducer (EMMPRIN), referred to as CD147, which recruits several types of metalloproteinases initially produced by surrounding stroma cells (Sameshima et al., 2000). Additionally, this mechanism activates pro-gelatinase A (proMMP-2) which is considered to be crucial for tumor progression. Generally, cells from brain tumor prefer a denser, more structuralized than normal, state of ECM and overexpress many of its components. Though this more compact microenvironment does not seem to impede the mobility of cancer cells, as they are able to synthesize a broad set of various matrix MMPs to enlarge the pore size in matrix to provide migration routes, and have other types of adaptations to overcome confined conditions (Nyagilo et al., 2017; Paul et al., 2017).

Apart from the prominent role in cancer cell migration and invasion, matrix components form a special type of microenvironment around normal neural stem cells (NSCs), called stem-cell niche. Meanwhile, tumors have a small subpopulation of cells with an exclusive capability of recapitulating all heterogeneity of cell subclasses within a tumor. These special cells, called cancer stem cells (CSCs), are critical to formation and progression of a tumor, and considered to be a major cause of therapeutic resistance and high relapse rate (Kondo et al., 2004).

The major difficulty stems from the fact, that some components are typical to niches of both CSCs and NSCs, however, there are few unique molecular signatures specifying each of these niches' types. Additionally, expression levels of various niche components may also vary (Table 2). For instance, expression levels of a typical ECM component of young PNNs, glycoprotein tenascin-C, are shown to directly correlate with the GBM malignancy grade and patients' prognosis (Chiquet-Ehrismann et al., 2014; Gulaia et al., 2018).

**Table 2:** Components of brain stem cell niches and their expression in normal and pathological condition (based on Reinhard et al., 2016, modified).

**ECM components**	**Neural stem cell (NSC) niche**	**Cancer stem cell (CSC) niche**
Aggrecan (ACAN)	+	–
Brevican	+	+++
Collagen	+	+
Fibronectin	+	+
Glypican-1	+	+++
Hyaluronic acid	+	+++
Laminin	+	+
Neurocan	+	+++
NG2 (neuroglia protein 2, NG2 proteoglycan)	+	+++
Perlecan	+	+
Phosphacan (DSD-1-PG)	+	+
Syndecan-2 (SDC2)	−	+
Tenascin-C	+	+++
Versican	+	+++

“*–”, not expressed; “+”, expressed in normal level; “+++”, overexpressed*.

Another example is neuroglia protein 2 (NG2) proteoglycan, also known as melanoma-associated chondroitin sulfate proteoglycan, is a membrane-anchored protein expressed by progenitor cells in the brain, especially oligodendrocyte progenitor cells. It is associated with ECM molecules, such as collagens, however NG2 is overexpressed in glioma (Wiranowska et al., 2006), and linked to migration capacity of cancer cells, since its expression levels in migratory cells are much higher than those of non-migratory cells (Wiranowska et al., 2006; Trotter et al., 2010).

Heparan sulfate proteoglycans (HSPGs) family member—syndecan-2 (SDC2), a membrane-anchored proteoglycan, was found to be expressed on high levels in brain tumor tissues, including gliomas, whereas never found in NSCs (Watanabe et al., 2006; Theocharis et al., 2010).

In addition, the expression of syndecan-1, glypicans, and HSPGs, which act as co-receptors for growth factors (FGF-2, PDGF, VEGF), stimulates angiogenesis and cell proliferation (Xiong et al., 2014) indispensable for tumor outgrowth.

Members of the laminin family construct NSC/CSC niches (Lathia et al., 2007). Laminins (together with collagen IV, nidogen, HSPGs, such as collagen XVIII and perlecan) mainly compose fractones, specialized ECM structures that contact NSCs (Kerever et al., 2007; Mercier, 2016). From the chemical perspective they are similar to basal membranes. Fractones, as described by Mercier, are ECM structural units, having a branched and fractal-like structure as they fill tight intercellular spaces, integrating NCSs with surrounding tissues, such as the subependymal layer. Moreover, laminin supports self-renewal of hippocampal neural stem/progenitor cells (Imbeault et al., 2009). The interaction of laminin and integrin α6 is shown to be important for the maintenance of NSCs/CSCs (Sun et al., 2008). However, in gliomas laminin α2 is produced not by glioma stem cells themselves but by non-stem tumor cells (Lathia et al., 2012).

It is widely known that CSPGs are highly expressed in the developing embryonic and adult brain as well as in glioma tissue. They influence cell mobility and axon growth and guidance (Lathia et al., 2012). Interestingly, in the adult healthy brain CSPGs exhibit inhibitory effects on stem cell migration. However, in glioma tissue upregulated CSPGs were reported to stimulate stem cell migration (Kearns et al., 2003; Sim et al., 2009). For example, CSPGs brevican and versican display significantly higher levels of expression in glioma. Both proteoglycans constitute networks comprising mesenchymal glioma-specific matrix molecules, which are not detectable in the healthy brain tissue (Reinhard et al., 2016). Brevican is especially enriched in astrocytoma and GBM, meanwhile it was demonstrated that its knockdown contributes to a reduction of late stage glioma tumor aggressiveness (Dwyer et al., 2014). Neurocan overexpression was also reported in glioma cells (Rauch, 2004; Varga et al., 2012). Other ECM components, such as hyaluronic acid, the adhesion molecule CD44 and tenascins interact with versican and stimulate brain tumor invasion. Whereas, aggrecan, another member of lecticans family, which is a typical component of the adult NSC niche alongside brevican (Mouw et al., 2014), was reported to be present only in normal NSC niches but not in CSC niches (Reinhard et al., 2016).

Thus, we can infer that many components of the normal brain ECM are overexpressed in tumor cells, which makes tumor ECM a special variety of cell microenvironment, more dense and structuralized than the rather amorphous normal brain ECM. Tumor cells, especially from glioma, demonstrate a significant set of adaptations to such confined conditions, and can successfully migrate and infiltrate into surrounding tissues. The ECM not only contributes to migration, but also plays an important role in cell survival, proliferation and differentiation processes not only in embryonic development but also in tumor growth.

## Interaction of Matrix Materials and Neoplastic Neural Cells

### Changes in Behavior of Neoplastic Neural Cells After Interaction With the Matrix

Currently, interaction of matrix materials and tumor cells of the neural tissue is being studied on a wide range of materials, both natural and artificial. Materials of natural origin are widely used, including (but not restricted to) those found in native ECM, such as type I collagen, hyaluronic acid, laminin, fibronectin, gelatin, alginate, as well as extracellular matrix extracts, for example, Matrigel—a laminin-rich extract of the extracellular matrix of mouse sarcoma. A significant variety of synthetic polymers that mimic extracellular matrix, such as polyethylene glycol (PEG), poly(lactic-co-glycolic acid) (PLGA), etc., are also proposed.

Development of new systems for cultivation of cancer cells is one of the methods for screening new anti-cancer drugs and studying cell-cell and the cell-ECM interactions under tumor conditions. Standard 2D culture of cells has a plurality of disadvantages and does not allow to reconstruct 3D tissue architecture that entails a discrepancy in the results of *in vitro* and *in vivo* experiments (Nyga et al., 2011; Alemany-Ribes and Semino, 2014).

It was shown that during invasion of the U87 glioma spheroid into Matrigel, spheroid cells had a compressive effect on the material, while invasive cells exerted a pulling effect on ECM (Gordon et al., 2003).

Further study of glioma cell interaction with ECM has shown that glioma cell migration is regulated by topographic signals that affect cell adhesion and gene expression (Agudelo-Garcia et al., 2011). It has been shown that spheroid invasion is facilitated in materials containing type I collagen due to an increase in the amount of fibers available for contact interaction. However, high concentrations of type I collagen inhibit cell growth inside the matrix (Kaufman et al., 2005). Cultivating cells with collagen materials, regardless of density of the fibers, enhances expression of genes associated with stemness, cell cycle, apoptosis, epithelial-mesenchymal transition, migration, and invasiveness. Wnt, Sonic Hedgehog, and Notch signaling pathways are involved in regulating these changes (Jia et al., 2018).

Matrices created exclusively from synthetic materials are mainly used to study their mechanical properties, since such properties can be varied in large range without changing the chemical composition. To achieve this, the base material is usually covered with a substrate that is part of the ECM. For example, a study using fibronectin-coated polyacrylamide hydrogels showed that cell migration depends on the rigidity of the substrate (Ulrich et al., 2009).

It was found that metalloproteases are critically necessary for cells' invasion into the material and its degradation. For example, when modifying PEG-based matrix material, leading to complete inaccessibility of material for degradation by these enzymes, U87 cells do not form protrusions and processes, while maintaining the ability to proliferate (Wang et al., 2017).

HA is a major component of the brain ECM, which is also believed to alter the phenotype of the invasive glioblastoma (Akiyama et al., 2001). Adding HA to a material made of gelatin and polyethylene glycol enhances malignancy of glioma cells (Pedron et al., 2013).

Pure HA hydrogel functionalized with cysteine-phenylalanine peptide residues does not affect the viability of primary GBM cells. A hydrogel may have different rheological properties, depending on the content of components. For drug delivery, Rowland et al. suggest using a hydrogel softer than brain tissue to enhance contact at the gel-tissue interface to facilitate drug diffusion. *Ex vivo* injection of an anticancer drug-loaded hydrogel into resected human tissue samples demonstrated effective gel delivery and graduate diffusion of the drug into the surrounding tissue (Rowland et al., 2018).

It has been established that invasion of U251 glioblastoma multiforme cells is enhanced in softer hydrogels, but is decreased in the presence of HA associated with the matrix. Blocking cell-matrix interactions of HA-CD44 reduces invasion even in hydrogels that do not have hyaluronic acid associated with the matrix. To stimulate invasion, glioblastoma cells produce free HA, thereby compensating for its lack in microenvironment (Chen et al., 2017).

Primary GBM cultures undergo significant genomic and transcriptional changes during cultivation, which should be taken into account in functional experiments and biomarker studies (Baskaran et al., 2018). Experiments on primary cultures are certainly important, but results obtained on different cells may not always be comparable even when using the same material. An experimenter, in fact, can record not the effect of the matrix itself, but the natural transformation of the molecular profile and functional activity of cells recently isolated from the natural microenvironment of tissues and placed *in vitro* culturing conditions. Therefore, it will be impossible to explicitly specify the influence of the matrix itself, and not the evolution of cell populations in a culture that always occurs.

### Changes in Signaling and Molecular Profiles of Neoplastic Neural Cells After Interaction With the Matrix

The above describes various ways of changing the extracellular matrix during glioblastoma progression. However, to date, little is known about the reverse effect of the extracellular matrix on cancer cells and development of tumors in response to changes in microenvironment.

3D culturing of U87, U251, and HS683 glioma cells using collagen showed an increase in expression of stem markers, such as CD133, Oct4, Sox2, and Nanog; cycle-related genes p21 and p27, genes related to the epithelial–mesenchymal transition (N-cadherin and vimentin) and invasion (MMP1, MMP2, MMP3, and MMP7), anti-apoptotic factors (PDL-1 and Livin), and a decrease in expression of pro-apoptotic factors, including caspases, poly (ADP-ribose) polymerase (PARP), and p53 (Jia et al., 2018).

In the study using poly-ε-caprolactone nanofibers as a substrate for cultivation, authors showed that glioma cell lines U87 and U251 demonstrate an extended morphology of cells migrating into the white matter tissue and are very sensitive to inhibition of myosin II in contrast to same cells cultivated on standard polystyrene. In this case, an increase in cell migration activity was achieved through the activation of STAT3 signaling, a known driver of glioma progression (Agudelo-Garcia et al., 2011).

An example of tumor growth activation in response to the extracellular matrix is shown in the work using chitosan-alginate materials (Kievit et al., 2014). After culturing cell lines U-87 MG and U-118 MG on these matrices cells acquired CD133^+^ phenotype and showed increased expression of Nestin and N-cadherin as compared to the conventional method of cultivation. Moreover, the growth of xenograft tumors in nude mice was significantly higher, when cells were used after cultivation with chitosan-alginate materials, which demonstrates importance of the effect of the matrix on the tumor cells.

Human glioblastoma cells U87, cultured on chitosan-polycaprolactone composite nanofibers, enhance migration and demonstrate activation of genes associated with invasion including β-catenin, Snail, STAT3, TGF-β, and Twist (Kievit et al., 2013).

Glioblastoma cells of patients with different expression of EGFR (EGFR wild type, EGFR^+^, and EGFRvIII) were studied in a culture model using matrix hyaluronic acid (HA) decorated with methacrylamide-functionalized gelatin (GelMA) (Pedron et al., 2017). It has been shown that phenotypically different tumors require extracellular matrix of different composition for the activation of key genes. Thus, for EGFR wild type cells, increased expression of VEGF, MMP-2, MMP-9 FN, and HIF-1 occurs at low concentrations of HA, and EGFR+ cells require a high concentration of HA. Moreover, authors showed that binding of CD44 to HA leads to the activation of EGFR in EGFR wild type and EGFR^+^, and the presence of HA in the matrix increases the resistance to tyrosine kinase inhibitor in EGFRvIII cells.

Not only the chemical composition of the extracellular matrix, but also its rheological characteristics, significantly moderates behavior of cancer cells. In the study using chitosan–HA scaffolds, authors showed that denser and stiffer materials lead to higher drug resistance in U-87 cells against temozolomide, increase the expression of chemoresistance markers (ABCG2), hypoxia inducible factor (HIF-1α), and invasion (CD44, MMP-2) (Erickson et al., 2018).

Despite development of methods for the treatment of malignant brain tumors, lifespan of patients with glioblastoma after surgical resection of the tumor rarely exceeds 15 months (Pinto et al., 2008). In this regard, in recent decades, the number of works aimed at developing alternative methods for drug delivery achieving direct effect on tumor cells based on biomaterials has increased.

However, currently there are very few works focuses on in-depth analysis of the action mechanisms of developed biomaterials on signaling pathways in glioblastoma cells.

Suppressing proliferation and reducing viability of human glioma cells U87 and U118 *in vitro* and *in vivo* resulting in reduction of tumor volume was observed in the work with the use of graphene plates (Jaworski et al., 2015). The authors of this work noted a high level of apoptosis in treated cells associated with the activation of proapototic gene CASP-3. In the study conducted with the use of hydrogels based on cellulose produced by bacteria *Komagataeibacter hansenii*, authors showed the ability of this material to attract and retain F98 glioblastoma cells; they offer to use this material together with chemoattractants for tumor cells for implantation in the area of tumor resection (Autier et al., 2019).

In addition to the anti-cancer effect, implantable materials should have a regenerating anti-inflammatory and neuroregenerative effect after surgical resection of the tumor. Currently, there are many works devoted to the regeneration of nervous tissue. Materials that promote regeneration are of both synthetic and natural origin. In the work with the use of polymeric ethyl acrylate and hydroxyethyl acrylate matrix *in vivo*, it was demonstrated that this material is populated after the implantation into the rat brain with cells of both glial and neuronal types (Martínez-Ramos et al., 2012). Scaffolds composed of electrospun poly-l/dl lactic acid (PLA70/30) nanofibers with Young's modulus around 140 MPa support the growth of glia and brain neurons and promote vascularization *in vitro* and *in vivo*, unlike non-aligned nanofibers with Young's modulus in the region of 40 MPa (Álvarez et al., 2014). Another study focuses on PEG-based hydrogels causing less astrocytic reaction in the injured brain compared to controls (Bjugstad et al., 2010). Poly ε-caprolactone matrices with brain derived neurotrophic factor (BDNF)-mimetics are able to be actively infiltrated with neuroblasts which further differentiate into neurons at the site of brain damage (Fon et al., 2014).

Numerous hydrogel types based on natural substances and their modifications are proposed today for regeneration of damage to both the spinal cord and the brain. In another study it was shown, that agarose hydrogels loaded with BDNF promote axon growth and demonstrate an anti-inflammatory effect in spinal cord lesions, compared to pure agarose (Jain et al., 2006). Alginate-based hydrogel with glial-derived neurotrophic factor causes functional restoration of the rat spinal cord hemisection model (Ansorena et al., 2013), and alginate-gelatin-based hydrogel has a stimulating effect on axonal growth in neurons of the cerebral cortex *in vitro* (Pawar et al., 2015).

Cancer cells respond not only to qualitative and quantitative changes in the microenvironment, but also to changes in mechanical properties of the extracellular matrix. Based on Hrapko's review of studies done over the past 50 years which focus on mechanical characteristics of the brain, it can be assumed that the values for G′ and G″ vary in the range from 100 to 104 Pa and from 20 to 1,000 Pa, respectively, depending on the method and conditions of measurement (Hrapko et al., 2008).

In the work with the use of polyacrylamide gels with a modulus of elasticity in the range from 300 Pa to 14 kPa, glioblastoma cells LN229 occupied the larger area, the harder polyacrylamide gels was on the cultivation surface. At the same time, normal astrocytes were less responsive to changes in the modulus of elasticity of the substrate. The authors did not discover any differences in mechanical properties of biopsy specimens of gliomas and healthy brain and suggested that increased stiffness due to vascularization and interstitial pressure in gliomas *in situ* may cause same reactions associated with increased substrate stiffness *in vitro* (Pogoda et al., 2014).

It is currently known that adult glioma aggression and patient prognosis may correlate with the stiffness of ECM (Miroshnikova et al., 2016). In general, non-tumor tissue shows the lowest level of ECM rigidity, while lower grade gliomas and GBM are more rigid; although there is heterogeneity between patients. Cell heterogeneity is consistent with the status of the isocytrate dehydrogenase-1 (IDH1) gene, since IDH1 is metabolic an enzyme which mutation is associated with greater survival without tumor progression (Gulaia et al., 2018). Most lower grade gliomas have a mutant IDH1, while GBM rarely carries such a mutation. Rigid GBM tumors with wild-type IDH1 have an impaired vasculature, leading to hypoxia, the emergence of necrotic foci, and signal transduction via hypoxia-induced factor-1α (HIF1α), a transcription factor that acts as the main effector of hypoxia. HIF1α binds directly to the tenascin-C promoter inducing its transcription (Miroshnikova et al., 2016). Tenascin-C modifies ECM by binding lecticans. Modification leads to the densification of tumor tissue relative to the normal brain, because lecticans are non-covalently bound to HA (Mouw et al., 2014). The IDH1 mutation reduces the susceptibility of glioma cells to hypoxia, which leads to a decrease in the production of HIF1α and tenascin-C, thereby reducing tumor aggressiveness.

Previously mentioned facts indicate that implantable materials can also be used as delivery vehicles for nerve stem cells and committed precursors of nerve cells. At the same time, the material simulates the biomechanical properties of the native tissue, supports the vital activity of the replacement cells and provides a contact guide for the directed growth of axons (Ghasemi-Mobarakeh et al., 2008; Yang et al., 2015). Moreover, materials are able to control cell fate, prevent the uncontrolled division of cells and determine the direction of their differentiation (Scanga et al., 2010; Wang et al., 2010).

Surgical removal of the tumor often leads to the post-operative complications including iatrogenic stroke (16.3 per 1,000 cases), hemorrhage or hematoma (10.3 per 1,000 cases) and other nervous system complications (8.2 per 1,000 cases). The mortality of the patients upon the occurrence of any surgical complications after resection of the tumor increases 4.4 times (De la Garza-Ramos et al., 2016). Thus, the issue of post-operative recovery and rehabilitation is quite acute. Based on cellular signaling and the difference in cell behavior depending on the stiffness of the matrix, we offer a two-step method in the treatment of gliomas. At the first step after removal of the tumor, the resection cavity should be filled with a relatively soft matrix with the addition of anti-cancer drugs inhibiting key oncogenic pathways, while the relatively soft matrix will help inhibit the growth of cancer cells because of its stiffness. After the proven success of the application of the first matrix, it can be replaced with a tougher matrix with the addition of the factors that stimulate the nervous tissue repair, as noted earlier, the matrices with relatively high rigidity stimulate the neurite outgrowth.

## Various Strategies for the Treatment of Glioblastoma Using Biocompatible Materials and Prospects for Their Use

When a matrix material is loaded with a drug, it is possible to significantly increase the life expectancy of patients after resection of glioblastoma and potentially eliminate it completely (Furnari et al., 2007). However, the *in vivo* implantation in the brain has several limitations:

1. Tumors in the brain are often localized near vessels and nervous tissue. In more than 90% of cases, relapse occurs at the edges of the resection cavity or within a few millimeters of the resection cavity.
2. There are several barrier systems in the brain that interfere with the delivery of cytotoxic drugs to tumor sites. Any damage to these barriers can lead to serious consequences.
3. The high degree of heterogeneity of GBM leads to variability in histopathology and the inability to reliably predict the response of the tumor to therapy.
4. The rapid and uncontrolled proliferation of GBM cells in combination with the above-mentioned properties allows the tumor to quickly develop resistance to apoptosis and chemoresistance, which usually leads to death for the patient.

To solve these problems and achieve the best outcome for a patient, many different strategies have been developed, like inventing barrier-penetrating drugs or changing the permeability of barriers in various ways (Bastiancich et al., 2016a, 2017; Zhao et al., 2018).

The main types of materials proposed for the treatment of GBM are:

1. Polymeric micelles are the particles of matrix formed by self-assembly of amphiphilic copolymers. They have a structure consisting of a hydrophilic shell and a hydrophobic core capable of absorbing and encapsulating poorly soluble drugs. Therefore, they are widely used as drug delivery systems in various therapeutic areas, including cancer treatment, in particular glioblastoma (Morshed et al., 2013; Yang et al., 2016).
2. Polymer nanoparticles comprising distinct type of material existing in several different variants, and many others are currently being developed. Their structure provides the ability to enclose drugs in particles or bind them to their surface. These materials, using various types of ligands, can be conjugated to magnetic nanoparticles, thus forming a more advanced type of matrix for targeted therapy (Bennet and Kim, 2014; Nam et al., 2018).
3. Lipid-based drug delivery systems (nanoparticles, micelles, liposomes) can be used to deliver drugs with poor water solubility. They can improve the bioavailability of such drugs and gradually release them over a long period of time. The great advantage of these systems over other drug carriers is their lower toxicity (Müller et al., 2002).
4. Hydrogels, constructing a three-dimensional (3D) polymer network, can absorb large amounts of water or biological fluid without dissolving the polymer due to its hydrophilic, but elastic structure. Therefore, polymeric matrices are able to encapsulate various biomacromolecules, similar to drugs, and gradually release them in a controlled manner over a long period of time (Bastiancich et al., 2016b).
5. Magnetic particles are nanoparticles made on the basis of gold, silver and gadolinium bringing up the most promising approach for the GBM treatment. A powerful magnetic field pulls drug particles out of suspension and delivers them to a localized site of the disease. Furthermore, magnetic particles are used to treat cancer by means of hyperthermia, as after reaching the tumor location, they are heated by an alternating magnetic field and thus thermally kill cancer cells (Maier-Hauff et al., 2007; Mahmoudi et al., 2018).

**Figure 2:**
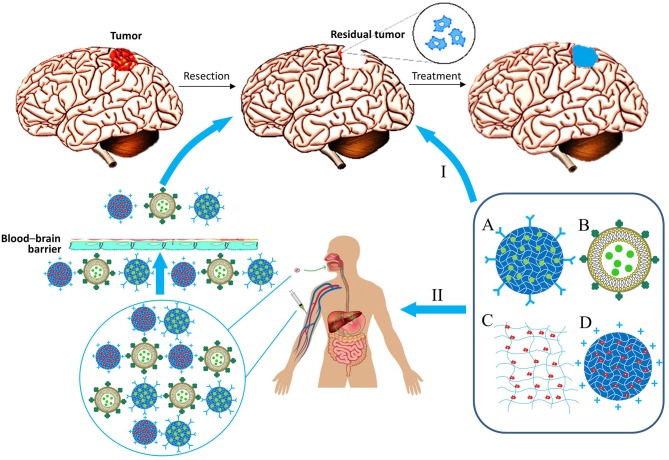
Biocompatible matrix for drug and cell delivery in glioblastoma treatment. Delivery ways: I, Local implantation in resection region; II, System administration. Types of material: A, Nanoparticle; B, lipid nanocapsule; C, Matrix; D, Nanoparticle with immobilized cells.

Materials consisting of small-sized particles (nanoparticles, micelles, and liposomes) can be delivered by injection into the bloodstream. Alternatively, it is possible to incorporate them into other gel materials, which can be injected directly into the site of tumor resection, or the delivery can be performed intragastrically (Wang et al., 2016). In this regard, matrix therapy can be largely divided into systemic and local (Figure 2).

Systemic therapy, as previously mentioned, can be accomplished by introducing the matrix intravenously or intragastrically with its subsequent delivery by the bloodstream across the blood-brain barrier (BBB). Many materials are capable of penetrating the BBB by themselves because of adsorptive-mediated transcytosis, when a particle is positively charged, so it binds to the surface of an endothelial cell and gets absorbed by it to be exocytosed on the surface of the lumen. However, ability of materials to overcome BBB can be further improved for effective treatment of brain cancers. Ability of nanoparticles/micelles to penetrate the blood-brain barrier is usually associated with the presence of certain peptides on their surface or antibodies specific for receptors on the surface of barrier cells (Pulgar, 2018). Some of the most important aforementioned receptors are the transferrin receptor, the insulin receptor, lipoprotein receptors, and folate receptors (Furtado et al., 2018). For instance, epirubicin liposomes modified with transferrin or tamoxifen significantly improved the efficacy of the anticancer drug in a rodent model of brain gliomas (Tian et al., 2010). Interestingly, it is feasible to bring carriers across the BBB by the cellular transport, e.g., the surface of the carrier is modified to improve phagocytosis by immune cells which then deliver particles through the BBB to the target location. This method can be further advanced by changing electrostatic and hydrophobic adsorption properties for phagocytosis activity improvement, ligand–receptor attachment, covalent coupling, and internalization (Stephan and Irvine, 2011). Physical and chemical properties of particles, such as size, shape, and type of material are also important for their effectiveness (Champion and Mitragotri, 2006; Albanese et al., 2012). Particles of materials can be covered with cellular membranes (e.g., erythrocyte membranes) for their “masking” to facilitate delivery through the BBB (Chai et al., 2017). With this delivery strategy, nano-sized particles are not required (Jain et al., 2003).

**Figure 3:**
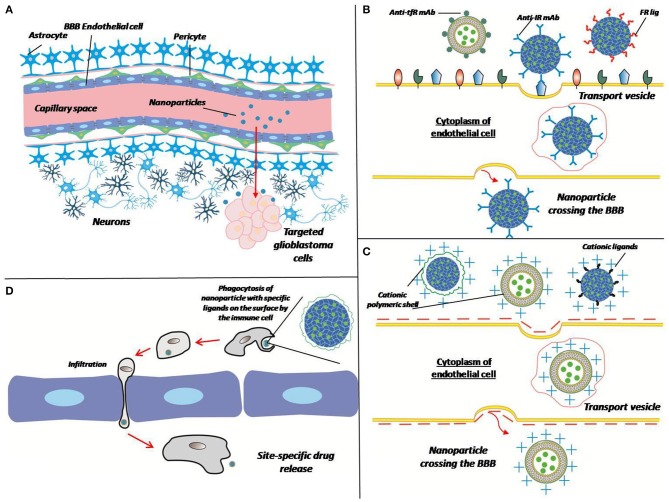
Different ways of crossing the BBB by the matrix. **(A)** Polymer particles enter the target site from the bloodstream by crossing the BBB. Overcoming the BBB can be performed in different ways **(B–D)**. **(B)** Receptor-mediated transcytosis: a nanoparticle can be coated with antibodies against transferrin receptor (Anti-tfR mAb), insulin receptor (anti-IR mAb), or folate receptor ligands (FR lig). Upon binding to the specific receptor nanoparticle is internalized in cytoplasm of endothelial cell thus crossing the BBB. **(C)** Adsorptive-mediated transcytosis: a nanoparticle conjugated with positively charged cationic ligands or cationic polymer shell associates with negatively charged membrane, thus induces membrane invagination and vesicle formation. **(D)** Cell-mediated transport: immune cell phagocytizes opsonized nanoparticle, crosses the BBB because of inflammation and release a drug at the site of tumor (inflammation).

All of the described methods of overcoming the BBB for delivery of matrices are summarized in the Figure 3.

However, systemic therapy has a number of disadvantages, modified from Srivastava et al. (2016):

1. Oral administration may result in degradation of the drug by the gastrointestinal enzymes or by its metabolization and inactivation in the liver.
2. Bloodstream administration of the matrix may trigger phagocytes and result in elimination of a large portion of particles.
3. Particles composed of hydrogels can quickly absorb the surrounding water possibly leading to premature drug release.
4. Utilization of nanoparticles implies specific shell and core construction, which should be immiscible, that further complicates microspheres production and design of their architecture.
5. The complexity of a reliable system development that could precisely control drug release from a depot.
6. The difficulty of creating successful designs for drug delivery through various barrier systems.
7. The systemic therapy requires the distinct way of matrix production inferring large volumes and high cost (Kim and Pack, 2006).

Thus, the most promising therapeutic strategy is local delivery of therapeutic agents into the brain cavity formed after the removal of a malignant neoplasm. This method does not have most of the drawbacks described above (matrix substances are introduced during the operation into the resection cavity, without affecting the CNS barriers and vessels). In addition, it helps to fill the gap in the course of the therapy between the operation and chemoradiation therapy (without impeding the process of operational wounds healing) (Bastiancich et al., 2017; Zhao et al., 2018). Further, we review the development of this strategy in more detail.

## Development of Implants for Local Chemotherapy

This strategy is based on the implantation of matrix materials (gels, nanoparticles, films, disks, rods, plates, etc.) and further gradual release of a drug into the surrounding brain tissues over a long period of time. During the release of the drug, matrix material should get decomposed to monomers suitable for metabolization or evacuation. If it is not biodegradable, there should be a possibility to remove matrix material. It must also gradually, over a long time period, release the drug in prescribed doses for effective action on tumor cells. The drug should have maximal possible effectiveness against cancer cells, good diffusion potential to prevent tumor relapse in remote areas, and should not cause chemoresistance in cancer cells. Importantly, the matrix itself and the drug should be hypoallergic to prevent possible therapy complications (Bastiancich et al., 2016a; Puente et al., 2018; Pinel et al., 2019).

This paper is focused mostly on hydrogels, because these polymer structures comprising the cutting-edge field of the most popular and promising tools for medical and biological manipulations. Hydrogels are injected directly into the brain after tumor resection via intracerebral implantation or intracerebroventricular injection.

Currently, Gliadel^®^ plates are the only implant approved by the US Food and Drug Administration (FDA) and the European Medical Agency (EMA). This is a biodegradable copolymer formed from 1,3-bis-(p-carboxyphenoxy) propane Saito(pCPP) and sebacic acid in a 20:80 ratio (polyfeprozan 20), impregnated with a chemotherapeutic drug BCNU (Carmustine) (Saito et al., 2017). The advantages of this implant are low systemic toxicity (reducing effects of gastrointestinal disorders, asthenia, fever and depression), gradual release of the drug over a period of time, and an increase in the long-term overall survival of patients (Xing et al., 2015). Disadvantages of this implant are serious local side effects, which include convulsions, intracranial hypertension, meningitis, swelling of the brain (causing repeated surgical intervention). The plates are poorly fixed and can migrate to other parts of the brain, which impairs wound healing. It was also demonstrated on model organisms that a larger portion of the drug is released in the first 5–7 days, and the range of the drug effect extended only barely beyond the polymer interface (3–6 mm during the first 7 days, 2–3 mm for the next 2 weeks); remote parts of the brain displayed insignificant concentrations of the drug. Although carmustine showed good results in treating tumor cells, temozolomide (TMZ) is more effective in treating malignant primary brain neoplasms, and therefore the choice of a drug for Gliadel^®^ is not considered to be the most optimal. In addition, only one third of GBM patients responded to carmustine treatment (Saito et al., 2017).

In connection with aforesaid facts, taking Gliadel^®^ errors into consideration, further developments are aimed at improving basic parameters of matrix and drug, as well as at increasing the functionality of these implants (Bastiancich et al., 2016a). Due to the fact that cancer cells go through various phases in tumor development and post-operational dynamics, which are characterized by shifts in cell microenvironment, they require different treatments strategies. Thus, we propose a novel approach to GBM treatment, described below.

**Figure 4:**
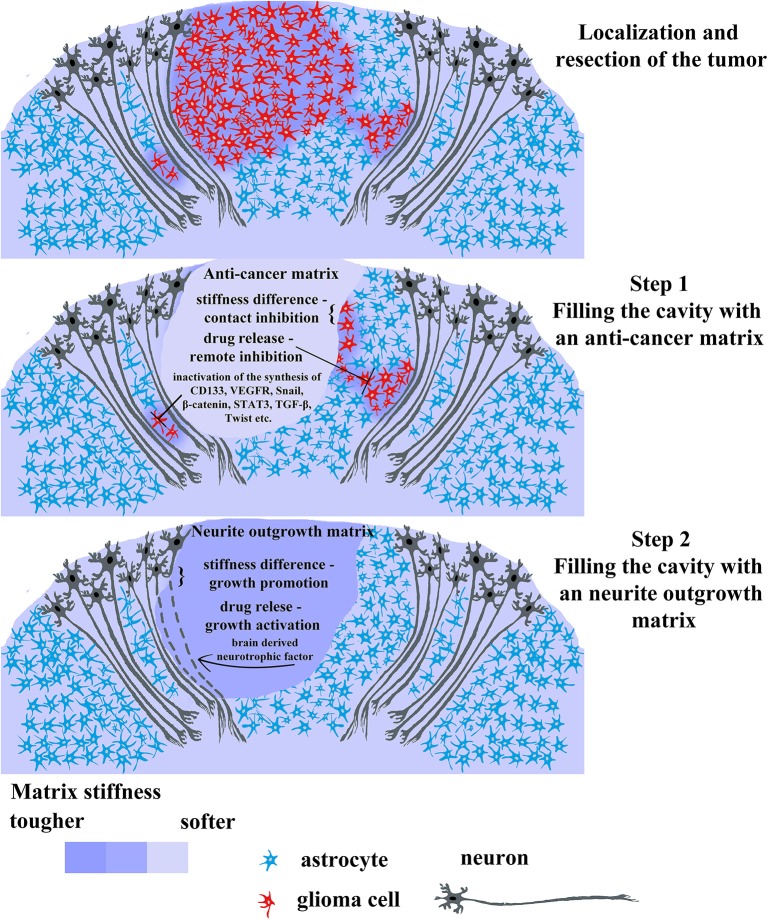
Promising therapeutic strategy for gliomas treatment using 2-step biomaterial insertion. The upper picture displays tumor consisting in red cells and surrounding tissue comprising normal glia cells (blue) and normal neurons (dark gray). The tumor has a primary lesion and diffusely infiltrating branches on the sides. After tumor localization and resection, the post-operative cavity can be filled with anticancer soft matrix (the middle picture) containing chemotherapeutic drugs capable of killing residual tumor cells and/or inhibiting main cancer associated pathways (CD133, VEGF, Snail, β-catenin, STAT, TGF-β, Twist). This soft matrix will prevent rapid regrowth of tumor cells as it physically occupies the cavity and release the chemotherapeutic drug killing infiltrated tumor cells left in brain parenchyma. Upon the first gel biodegradation and complete drug release, the cavity will be filled by the filled with the second denser matrix facilitating wound healing and neuron axon regrowth (the lower picture). The last step is necessary for avoiding scar formation and faster rehabilitation from prospective neurological complications after the surgery.

We suggest that the scientific community and cancer therapists draw the attention to the possibility of developing a two-stage approach to the treatment of particular glioma cases. Taking into account advantages of 3D cell cultivation on the one hand, and the heterogeneity of gliomas on the other, it can be concluded that there is a demand for development of a biocompatible matrix, aimed at the direct delivery of anticancer drugs, blocking key molecular participants in carcinogenesis, and regeneration of healthy nervous tissue after tumor resection. These two opposite processes can be applied in two successive stages (Figure 4):

1. Filling the post-operative cavity with the matrix possessing a relatively low modulus of elasticity loaded with anticancer drugs, preventing hypoxia, and aiming at inhibiting key pro-oncogenic pathways (inactivating synthesis of cd133, vegfr, β-catenin, snail, stat3, tgf-β, twist etc.).
2. Substitution for a matrix with a higher modulus of elasticity, loaded with brain derived neurotrophic factor or its analogs enabling the sprouting of nerve cells.

Carcinogenesis is known to be frequently associated with the development of cognitive impairments [from 25 to 44% depending on the particular impairment (Armstrong et al., 2006)]. Some cognitive dysfunctions, such as poor verbal memory and executive dysfunction can either persist after surgery (Veretennikoff et al., 2017) or undergo remission to some extent (Dhandapani et al., 2016). The two-stage approach is aimed at improving the quality of life for such patients; however, the patient's safety and the increathe of his life expectancy should remain the priority in the fight against tumors in general and gliomas in particular. In this context, if the risk of matrix delivery to the tumor site located in a hard-to-reach region exceeds the potential positive therapeutic effect, then the two-stage approach should be abandoned. If the tumor is located in a surgically “safe” area, then the first stage can be implemented after the standard chemotherapeutic course; e.g., the introduction of a matrix with the relatively low elastic modulus, that is loaded with antitumor drugs and/or cytostatics against key oncogenic pathways. We propose an elastic modulus ≤1,000 Pa for the first stage, since this elasticity does not stimulate the outgrowth formation and is able to suppress cell proliferation (Klein et al., 2009), while for the second stage we recommend an elasticity modulus ≥1,000 Pa to simulate neurite regeneration (Klein et al., 2009; Pogoda et al., 2014). The duration of the first stage should be approximated in accordance with the anticancer drug release time and the time of matrix biodegradation (in case of the biodegradable matrix), for instance, Gliadel^®^ wafers completely degrade during 6–8 weeks (Wu et al., 1994). Thus, we suggest calculating the first stage time in accordance with the duration of the matrix biodegradation, however, the rapid and uneven release of the loaded drug (Grossman et al., 1992) could be the reason to adjust the calculation. The frequency of relapse after tumor removal is quite high—around 50%, and the most frequently it occurs within the resection cavity on the average from 6–9 (Mallick et al., 2016) to 17 months (Konishi et al., 2012). In case of using a non-degrading matrix at the first stage, it should be removed from the resection cavity and replaced with a matrix with a higher elastic modulus loaded with the brain derived neurotrophic factor or its analogs. Since the first stage is specifically aimed at protecting the patient from a relapse as well as at destroying remained proliferating cancer cells near the resection cavity, we suggest that a 6-months period between the stages will be optimal and should be taken to monitor the patient for a possible relapse. There is a certain risk of relapse initiation by a matrix loaded with the brain-derived neurotrophic factor or its analogs due to the remaining “quiescent cancer stem cells,” however, there is a theory suggesting that proliferative stimulation of these cells can increase their sensitivity to anticancer drugs (Gulaia et al., 2018). On the one hand, the first stage is aimed specifically at protecting the patient from relapse and killing the remaining cancer cells near the resection cavity, on the other hand, the second stage employs the matrix stimulating the nerve tissue growth and has antagonistic properties compared to the first stage matrix. In addition to certain requirements for elasticity, the second stage material should have specific biodegradation rate optimal for nerve tissue regeneration. Many experimental data available for tissue regeneration in the stroke models prompt us to suggest a period of 3–4 months for the second stage (Cook et al., 2017; Ghuman et al., 2018; Nih et al., 2018).

Thus, we propose a unique approach employing the usage of two matrices subsequently, in the way, that first matrix prevent the tumor regrowth as the absence of empty space and prolonged drug release will hinder this process, while the second matrix possessing denser structure will allow neurons to regrow their damaged axons and dendrites. Therefore, the latter matrix will be implemented for faster recapitulation of neurological consequences from surgery.

In addition, further we review the latest trends in the creation of matrices and implants for the treatment of GBM.

## Various Polymer Matrices Used for Drug Delivery

### Fully Synthetic-Based Matrices

Drug delivery in case of GBM could be hindered by BBB preventing therapeutic drugs and small molecules reaching the tumor growth site. The polymeric matrices are promising in terms of biocompatibility and prolonged drug delivery time. This group includes polymeric materials that are synthesized from various monomers and have little or no effect on cell niches.

#### PLGA-Based Matrices

PLGA is a biodegradable and biocompatible polymer that is used in a number of FDA-approved therapeutic devices (grafts, sutures, implants, prosthetic devices, surgical sealant films, etc.). PLGA biodegradable nanofibers can release high concentrations of vancomycin for more than 8 weeks in the cerebral cavity of rats (Tseng et al., 2013). A number of publications reported that it can be used for the treatment of brain tumor as an alternative drug delivery system (Bagó et al., 2016; Ramachandran et al., 2017; Zhao et al., 2018). Modification of PLGA with copolymer Poloxamer 188 renders it the capability of penetrating BBB more effectively (Malinovskaya et al., 2017). Despite the large number of studies, systemic toxic effect of the drugs delivered by the PLGA nanoparticles remains not fully understood and requires further research.

One of the most effective and easy to produce of the biodegradable gel matrices for the delivery of TMZ was developed in 2008 by Akbar et al. It consists of PLGA plasticizers (40:60) loaded with temozolomide; the plasticizers themselves consist of acetyl triethyl citrate and triethyl citrate (30:30). The authors developed a surgical resection model of intracranial glioma C6-GFP in rats to test this matrix *in vivo*. Introduction of implants loaded with 30% TMZ resulted in a decrease of the tumor size by 94%, accompanied by a slight decrease in animal weight and neurological activity. The implants used demonstrated complete biodegradability and efficiency, and continued drug delivery to the peritumoral areas beyond a 15-days period (Akbar et al., 2009).

In 2011, Ozeki et al. developed microspheres containing thermoreversible gelation polymer TGP hydrogel with camptothecin (CPT), and evaluated its therapeutic efficacy (comparison of survival) on the C6 glioma model and a tumor resection model. Treatment with CPT/PLGA/TGP showed significant survival compared with the untreated rats (26 vs. 18 days, respectively). Similar therapeutic effects were observed in groups that received only CPT/PLGA/TGP and surgical tumor resection plus CPT/PLGA/TGP, but some long-term survivors (>60 days) were observed in this latter group, which means that combination therapy may be a good strategy for this hydrogel. At the same time, ICG fluorescence in the ICG/PLGA/TGP remained localized for 28 days after injection. Moreover, neither significant body weight loss nor aggressive immune response were observed (Ozeki et al., 2012).

#### PEG-Based Matrices

PEG as a component of hydrogels enhances the absorbance of water-soluble substances. Presence of a controlled pore size makes it a suitable carrier for targeted drug delivery. The potential of this material in treatment of spinal injuries is being studied (Isa et al., 2015). Mostly, PEG is used in combination with PLGA.

Another interesting implant based on PLGA and PEG is OncoGel™. This hydrogel was tested in 2008 by Tyler et al. as an adjuvant for radiotherapy on an intracranial model. OncoGel™ consists of Cremophor^®^ EL-based paclitaxel immersed in ReGel™, which is a copolymer of PLGA and PEG. With the change in temperature from room to normal body, the viscosity of the matrix is shifted to high thus forming a gel once inside body. Viscosity change comes along with the modifications in other properties, e.g., matrix turns into biodegradable gel insoluble in water with controlled release of the drug. Tests on model organisms showed an increase in median survival with 37.5% of long-term survivors and the absence of toxicity and any pathological changes. At the same time, ReGel loaded with paclitaxel is biodegradable and releases paclitaxel at a constant rate for ~50 days (Tyler et al., 2010).

In 2010, the thermally reversible gel-forming polymeric hydrogel TGP (Mebiol™ Gel) was developed. This matrix exhibit unique properties as it has a liquid form at room temperature, while become a gel once the temperature rises to normal body one. It consists of PEG conjugated with thermosetting polymeric poly-N-isopropylamide. TGP is biocompatible, non-cytotoxic, and completely pathogen-free. Tests were performed on a human GBM subcutaneous xenograft model demonstrating significant inhibition of tumor growth during drug encapsulation. Drug releases from TGP-dox combined with sphere- dox or lipo-dox lasted for 32 and 38 days, respectively (Arai et al., 2010).

An interesting representative of a photopolymerizable hydrogel is a type of injectable matrix described in 2015 by Fourniols et al. This matrix consists of a mixture of PEG dimethacrylate (PEG-DMA) and water (75:25), as well as 0.5% Lucirin-TPO^®^ as a photoinitiator. When this mixture is exposed to 400 nm light for 15 s, the matrix quickly changes its properties (<2 min) and acquires a viscosity modulus of around 10 kPa. Studies conducted in model animals showed that the hydrogel does not cause apoptosis in the brain of mice and has no impact on the activation of microglia, while the antitumor effectiveness in mice treated with photopolymerized hydrogel was significantly enhanced compared to conventional drug delivery (Fourniols et al., 2015).

In 2018, an injectable photopolymerizable PEG-DMA hydrogel was developed and characterized by Zhao et al. It was synthesized using a modified emulsion diffusion procedure containing exclusively photopolymerizable PEG-DMA loaded with Paclitaxel (PTX) PLGA-nanoparticles. *In vivo* tolerability studies showed that implantation of hydrogel into brains of healthy mice was well tolerated for the short time period (1 week) as well as for the long period (4 months). Studies of *in vivo* antitumor efficacy carried out on a clinically relevant tumor model (orthotopic model of U87 resection in mice) showed that PTX PLGA-nanoparticles significantly prolonged the survival of mice, and 50% of mice were alive 5 months after tumor implantation (Zhao et al., 2018).

#### Other Matrices

In another study, the matrix comprising doxorubicin elution granules was evaluated for safety and efficacy on the 9L glioma model. This matrix consists of microspheres composed of modified polyvinyl alcohol with sulfonate groups mixed with the 0,6% alginate solution. Additionally, one can choose a therapeutic agent for this matrix, for example, between irinotecan, topotecan and mitoxantrone. Studies on model organisms showed significant results compared with untreated animals in terms of survival (44, 54, and 26 days, respectively), accompanied by the absence of significant immune response and weight loss. Also, studies have shown that doxorubicin was still released from the beads at 90 days point after implantation (Vinchon-Petit et al., 2010).

A notable example of a pH/temperature-sensitive matrix is a magnetic nanogel, tested in 2010, which contains contrast media for MR and fluorescence imaging. This matrix consists of N-isopropylacrylamide-co-acrylic acid nanogel loaded with superparamagnetic iron oxide (SPIO) nanoparticles that have been conjugated with Cy5,5-lactoferrin. This implant was tested for the treating of rat glioma C6 tumors *in vivo*. The grafted fluorophore Cy5,5 is responsible for fluorescent imaging, SPIO helps to detect nanoparticle accumulations in brain tumors, while lactoferrin is a ligand for protein 1 linked to a low-density lipoprotein receptor which is overexpressed in GBM. Likewise this matrix was proved not to cause inflammation and significant weight loss (Xie et al., 2011; Jiang et al., 2013).

### Matrices Based on Natural Compounds

Natural matrices contains polymeric materials in which the matrix backbone is partially or completely composed of substances isolated from living organisms or synthesized on the basis of natural compounds.

Chitosan is a 1,4-linked 2-amino-2-deoxy-β-D-glucan which can be cleaved by lysozyme in animals, thus possessing the advantage of natural *in vivo* biocompatibility and biodegradability. Xie et al. synthesized stearic acid with grafted chitosan and investigated the physicochemical characteristics of obtained micelles. The material showed promising results in *in vivo* studies of glioma C6 treatment (Xie et al., 2012).

Alginates are alginic acid salts. Unlike the insoluble alginic acid, potassium and sodium alginates form colloidal solutions in water. Addition of sodium alginate water solution to solutions containing calcium ions (e.g., CaCl) leads to the formation of insoluble calcium alginate gels. Alginates are non-toxic, and also highly compatible with microenvironment in the brain (Lee and Mooney, 2012), so its microcapsules can be used to immobilize and deliver peptides with anticancer activity to the brain (Read et al., 2001).

Dextran is a branched glucose polymer with an average weight of chains ranging from 3 to 20,000 kDa. The main chain consists of molecules linked by the α-1.6 bond, and side branches are attached by α-1.3 bonds. When used as matrices, dextrans are coated with iron oxide nanoparticles to make its aqueous solution a stable dispersion systems. Currently, dextran platforms are being developed for the controlled release of doxorubicin in the treatment of GBM. Preliminary studies have shown that such matrices are superior to polylactic acid based materials in preventing the recurrence of glioblastoma in rats (Graham-Gurysh et al., 2018).

In 2014, a matrix consisting of cytarabine-loaded phospholipid vesicles was described by Qi et al., which composed of a gel-like semi-solid dispersion of phospholipids. The peculiarity of this matrix is the high resistance to autoclaving, which meets one of the main requirements of hydrogels for the use in the treatment of brain cancers: sterility. The phospholipid vesicles implant in animal models was shown to release a drug (cytarabine) for at least 28 days with a good biodistribution profile and penetration depth of a drug after intracerebral injection. In addition, no implant rejection was observed (Qi et al., 2012, 2014).

An excellent example of a hydrogel implant based on lipid nanocapsules was described in 2015 by Aparicio-Blanco and Torres-Suárez (Aparicio-Blanco and Torres-Suárez, 2015). This matrix is uniquely formed byx lipid nanocapsules (LNC) in combination with GemC12 (Gemcitabine); besides that gel does not contain any polymeric components. The formation of this hydrogel occurs due to location of GemC12 at the interface of the oil-water LNC, which ensures the formation of cross-linking H-bonds between the drug fragments and immobilization of the aqueous phase. Compared to previously mentioned varieties of hydrogels, the advantage of this system is in the structure simplicity as the gel decomposition results in release of only GemC12-LNC, because it contains no other components (synthetic or natural polymers, gelling agents, external irritants), which reduces the risk of side effects. *In vivo* studies showed that this system showed is well-tolerated in the brain of mice, both in short (1 week) and long (2 and 6 months) experiments (Bastiancich et al., 2016b, 2017). The degradation of the hydrogel corresponded to the sustained release of the drug, which lasted over 1 month. Intratumoral administration of this hydrogel in a human orthotopic xenograft model of a person with GBM showed a significant increase in survival compared with the control (Bastiancich et al., 2018).

The melittin-containing peptide hydrogel Melittin-RADA 32 Indocyanine green described by Jin et al. in 2017 also raised significant interest. It was constructed from the RADA32-melittin fusion peptide in the presence of 0.9% NaCl and encapsulated in the hydrogel, with indocyanine green added. *In vivo* experiments with this hydrogel showed excellent results in visualizing residual tumor cells. Three hours after the hydrogel administration, Magnetic resonance imaging analysis showed significant signals in tumors, thus it is possible to distinguish diffused tumor cells left after surgery. Furthermore, this hydrogel is able to release the drug in required doses and protect the implant itself from rapid decomposition or elimination. However, melittin has non-specific cytotoxicity and hemolytic activity, which limits its therapeutic use (Jin et al., 2017).

The most unusual implant is a chitosan-based hydrogel capable of releasing temozolomide while including radioactive iodine isotopes. This implant is applied for local complex therapy providing chemo- and radiation therapy simultaneously. At the time of writing, the first tests of this hydrogel were carried out, but there was no extensive data. However, the release of drug was negligible after 42 days, whereas the TMZ was completely released over the first 48 h (Puente et al., 2018). Nevertheless, the authors believe that this trend is promising due to the fact that post-operative systemic therapy has a complex effect, and therefore local therapy should be more convenient. However, at the moment there is no data on the survival of the model objects as well as the possible drawbacks of this hydrogel.

## Conclusions

In recent years, targeted delivery systems have been developed for various therapeutic agents, such as chemotherapy drugs, stem or committed cells, which all can be used in the treatment of gliomas and provide a substantial improvement in its prognosis (Nakamizo et al., 2005; Lee et al., 2011; Hu et al., 2013). Carriers are materials of different nature, which to a greater or lesser extent have such advantages as custom mechanical properties, good biocompatibility, conventional way of matrix introduction (during operation), as well as the ability to deliver various biologically active substances and drugs.

In the treatment of brain tumors hydrogels can be employed for the effective delivery of chemotherapeutic agents while do not require highly invasive procedures. They proved to be unique platforms for local and systemic methods of drug delivery due to their high biocompatibility, biodegradability, and the presence of active functional groups. Combination of nanoparticles and hydrogels bringing up a possibility of creating material capable of crossing the BBB and effectively detecting and destroying tumor cells without harming healthy brain cells.

Hydrogels can also be used for targeted delivery to tumor cells and the delivery of highly toxic drugs. For example, when encapsulating immunostimulating drugs in a hydrogel, it becomes possible to deliver them to the resection cavity without the need to cross the BBB and with the possibility of their gradual release. It is also possible to reduce the total damage to the patient's body by encapsulating toxic drugs, such as platinum nanoparticles. However, the best results can be achieved by encapsulating these drugs in modified nanoparticles, which will be placed in the resection cavity in the hydrogel. This approach will minimize the disadvantages of systemic delivery of these drugs, almost completely reduce the toxic harm to the patient's body, but at the same time increase the therapeutic effectiveness of these drugs (Xiao et al., 2018; Feng et al., 2019; Yu et al., 2019).

Synthetic polymers are widely used as components of systems for drug delivery due to their well-known chemical and structural characteristics, as well as the ability to control and easily adjust the physico-chemical properties of the matrix. However, synthetic materials do not display biological activity and lack the ability to fully imitate the native extracellular matrix even after modification by adhesive sites. Unfortunately, the degradation of synthetic material can lead to release of cytotoxic or inflammatory small molecules (Stoppel et al., 2015).

Recently, growing interest has been raised to the field of matrices consisting partially or entirely of natural materials, due to their ability to influence cell proliferation at the edges of the resection cavity. This effect can reduce the risk of tumor recurrence from residual cells that were omitted during surgery (Huynh et al., 2009).

Natural materials are derived from substances of plant or animal origin. After purification and preparation for *in vivo* use they usually do not cause an undesirable or unexpected immune response proposing a source for biocompatible and often biologically active matrix capable of integration with the surrounding native tissue (Lee et al., 2014). In addition, the products of natural material degradation are usually more biologically compatible, metabolically acceptable, and less toxic than synthetic analogs. Disadvantages of biomaterials from natural origin are their chemical heterogeneity in combination with high dispersion that, as a result, cause variability of structure, mechanical properties, and degradation rates between different batches of the same material. Despite variability, biopolymer matrix materials of natural origin have been successfully applied clinically for the restoration of soft (skin and muscles) (Später et al., 2018) and hard tissues (bone) (Giuliani et al., 2014).

A lot of studies reviewed in this paper reported significantly increased efficiency of tumor growth inhibition as a result of utilizing matrix materials after resection, as well as synergistic effect that can be achieved through a combination of several chemical agents or therapeutic approaches. To solve all vital problems arising during GBM treatment (precise tumor localization, eliminating delays in the course of treatment, prevention of relapse), it is necessary to further study multifunctional implants, since they are capable of passing over the maximum number of obstacles with minimal intervention in the human brain.

Thus, promising hydrogel-based drug co-delivery systems will make a significant contribution to cancer treatment and human health.

## Author Contributions

AB, ST, NS, MG, GM, VG, AK, and VK wrote the manuscript. AB, GM, ST, and NS drew the figures. All authors approved the final manuscript.

### Conflict of Interest

The authors declare that the research was conducted in the absence of any commercial or financial relationships that could be construed as a potential conflict of interest.

## Abbreviations

BBB: blood-brain barrier
BDNF: brain-derived neurotrophic factor
CNS: central nervous system
CPT: Camptothecin
CSC: cancer stem cell
CSPG: chondroitin sulfate proteoglycan
DMA: dimethacrylate
ECM: extracellular matrix
EGFR: epidermal growth factor receptor
FDA: Food and Drug Administration
GAG: glycosaminoglycan
GBM: glioblastoma multiforme
GelMA: gelatin methacryloyl
GFP: green fluorescence protein
HA: hyaluronic acid
HSPG: heparan sulfate proteoglycan
LNC: lipid nanocapsules
MMP: matrix metalloproteinase
NSC: neural stem cell
PEG: polyethylene glycol
PLA: polylactic acid
PLGA: poly(lactic-co-glycolic acid)
PNN: perineuronal net
PTX: Paclitaxel
SPIO: superparamagnetic iron oxide
TGP: Thermo-Reversible Gelation Polymer
TMZ: temozolomide
VEGF: vascular endothelial growth factor.

## References

[B1] Agudelo-Garcia P. A. De Jesus J. K. Williams S. P. Nowicki M. O. Chiocca E. A. Liyanarachchi S.. (2011). Glioma cell migration on three-dimensional nanofiber scaffolds is regulated by substrate topography and abolished by inhibition of Stat3 signaling. Neoplasia 13, 831–840. 10.1593/neo.11612 21969816 PMC3182275

[B2] Akbar U. Jones T. Winestone J. Michael M. Shukla A. Sun Y.. (2009). Delivery of temozolomide to the tumor bed via biodegradable gel matrices in a novel model of intracranial glioma with resection. J. Neurooncol. 94, 2031–2212. 10.1007/s11060-009-9857-9 19337695

[B3] Akiyama Y. Jung S. Salhia B. Lee S. Hubbard S. Taylor M.. (2001). Hyaluronate receptors mediating glioma cell migration and proliferation. J. Neurooncol. 53, 115–127. 10.1023/A:1012297132047 11716065

[B4] Albanese A. Tang P. S. Chan W. C. (2012). The effect of nanoparticle size, shape, and surface chemistry on biological systems. Annu. Rev. Biomed. Eng. 14, 1–16. 10.1146/annurev-bioeng-071811-150124 22524388

[B5] Alemany-Ribes M. Semino C. E. (2014). Bioengineering 3D environments for cancer models. Adv. Drug Deliv. Rev. 79–80, 40–49. 10.1016/j.addr.2014.06.004 24996134

[B6] Álvarez Z. Castaño O. Castells A. A. Mateos-Timoneda M. A. Planell J. A. Engel E.. (2014). Neurogenesis and vascularization of the damaged brain using a lactate-releasing biomimetic scaffold. Biomaterials 35, 4769–4781. 10.1016/j.biomaterials.2014.02.051 24636215

[B7] Ansorena E. De Berdt P. Ucakar B. Simón-Yarza T. Jacobs D. Schakman O.. (2013). Injectable alginate hydrogel loaded with GDNF promotes functional recovery in a hemisection model of spinal cord injury. Int. J. Pharm. 455, 148–158. 10.1016/j.ijpharm.2013.07.045 23916821

[B8] Aparicio-Blanco J. Torres-Suárez A. I. (2015). Glioblastoma multiforme and lipid nanocapsules: a review. J. Biomed. Nanotechnol. 11, 1283–1311. 10.1166/jbn.2015.2084 26295134

[B9] Arai T. Benny O. Joki T. Menon L. G. Machluf M. Abe T. (2010). Novel local drug delivery system using thermoreversible gel in combination with polymeric microspheres or liposomes. Anticancer Res. 30, 1057–1064. 20530409

[B10] Armstrong L. Hughes O. Yung S. Hyslop L. Stewart R. Wappler I.. (2006). The role of PI_3_K/AKT, MAPK/ERK and NFκβ signalling in the maintenance of human embryonic stem cell pluripotency and viability highlighted by transcriptional profiling and functional analysis. Hum. Mol. Genet. 15, 1894–1913. 10.1093/hmg/ddl112 16644866

[B11] Autier L. Clavreul A. Cacicedo M. L. Franconi F. Sindji L. Rousseau A.. (2019). A new glioblastoma cell trap for implantation after surgical resection. Acta Biomater. 84, 268–279. 10.1016/j.actbio.2018.11.027 30465922

[B12] Bagó J. R. Pegna G. J. Okolie O. Mohiti-Asli M. Loboa E. G. Hingtgen S. D. (2016). Electrospun nanofibrous scaffolds increase the efficacy of stem cell-mediated therapy of surgically resected glioblastoma. Biomaterials 90, 116–125. 10.1016/j.biomaterials.2016.03.008 27016620 PMC5376347

[B13] Baskaran S. Mayrhofer M. Kultima H. G. Bergström T. Elfineh L. Cavelier L.. (2018). Primary glioblastoma cells for precision medicine: a quantitative portrait of genomic (in)stability during the first 30 passages. Neurooncology 20, 1080–1091. 10.1093/neuonc/noy024 29462414 PMC6280139

[B14] Bastiancich C. Bianco J. Vanvarenberg K. Ucakar B. Joudiou N. Gallez B.. (2017). Injectable nanomedicine hydrogel for local chemotherapy of glioblastoma after surgical resection. J. Control. Release 264, 45–54. 10.1016/j.jconrel.2017.08.019 28830791

[B15] Bastiancich C. Danhier P. Préat V. Danhier F. (2016a). Anticancer drug-loaded hydrogels as drug delivery systems for the local treatment of glioblastoma. J. Control Release 243, 29–42. 10.1016/j.jconrel.2016.09.034 27693428

[B16] Bastiancich C. Lemaire L. Bianco J. Franconi F. Danhier F. Préat V.. (2018). Evaluation of lauroyl-gemcitabine-loaded hydrogel efficacy in glioblastoma rat models. Nanomedicine. 13, 1999–2013. 10.2217/nnm-2018-0057 30204064

[B17] Bastiancich C. Vanvarenberg K. Ucakar B. Pitorre M. Bastiat G. Lagarce F.. (2016b). Lauroyl-gemcitabine-loaded lipid nanocapsule hydrogel for the treatment of glioblastoma. J. Control Release 225, 283–293. 10.1016/j.jconrel.2016.01.054 26829100

[B18] Bellail A. C. Hunter S. B. Brat D. J. Tan C. Van Meir E. G. (2004). Microregional extracellular matrix heterogeneity in brain modulates glioma cell invasion. Int. J. Biochem. Cell Biol. 36, 1046–1069. 10.1016/j.biocel.2004.01.013 15094120

[B19] Bennet D. Kim S. (2014). Polymer Nanoparticles for Smart Drug Delivery. London: Intechopen.

[B20] Bilozur M. E. Hay E. D. (1988). Neural crest migration in 3D extracellular matrix utilizes laminin, fibronectin, or collagen. Dev. Biol. 125, 19–33. 10.1016/0012-1606, 90055-3 3275424

[B21] Bjugstad K. B. Lampe K. Kern D. S. Mahoney M. (2010). Biocompatibility of poly(ethylene glycol)-based hydrogels in the brain: an analysis of the glial response across space and time. J. Biomed. Mater. Res. Part A 95, 79–91. 10.1002/jbm.a.32809 20740603

[B22] Bouterfa H. Janka M. Meese E. Kerkau S. Roosen K. Tonn J. C. (1997). Effect of changes in the CD44 gene on tumour cell invasion in gliomas. Neuropathol. Appl. Neurobiol. 23, 373–379. 10.1111/j.1365-2990.1997.tb01311.x 9364462

[B23] Chai Z. Hu X. Wei X. Zhan C. Lu L. Jiang K.. (2017). A facile approach to functionalizing cell membrane-coated nanoparticles with neurotoxin-derived peptide for brain-targeted drug delivery. J. Control Release 264, 102–111. 10.1016/j.jconrel.2017.08.027 28842313

[B24] Champion J. A. Mitragotri S. (2006). Role of target geometry in *phagocytosis*. Proc. Natl. Acad. Sci. U.S.A. 103, 4930–4934. 10.1073/pnas.0600997103 16549762 PMC1458772

[B25] Chen J. E. Pedron S. Harley B. A. C. (2017). The combined influence of hydrogel stiffness and matrix-bound hyaluronic acid content on glioblastoma invasion. Macromol. Biosci. 17:1700018. 10.1002/mabi.201700018 28379642 PMC5555785

[B26] Chiquet-Ehrismann R. Orend G. Chiquet M. Tucker R. P. Midwood K. S. (2014). Tenascins in stem cell niches. Matrix Biol. 37, 112–123. 10.1016/j.matbio.2014.01.007 24472737

[B27] Cook D. J. Nguyen C. Chun H. N. Llorente I. L. Chiu A. S. Machnicki M.. (2017). Hydrogel-delivered brain-derived neurotrophic factor promotes tissue repair and recovery after stroke. J. Cereb. Blood Flow Metab. 37, 1030–1045. 10.1177/0271678X16649964 27174996 PMC5363479

[B28] Cuddapah V. A. Robel S. Watkins S. Sontheimer H. (2014). A neurocentric perspective on glioma invasion. Nat. Rev. Neurosci. 15, 455–465. 10.1038/nrn3765 24946761 PMC5304245

[B29] De la Garza-Ramos R. Kerezoudis P. Tamargo R. J. Brem H. Huang J. Bydon M. (2016). Surgical complications following malignant brain tumor surgery: an analysis of 2002-2011 data. Clin. Neurol. Neurosurg. 140, 6–10. 10.1016/j.clineuro.2015.11.005 26615463 PMC4750489

[B30] Delpech B. Maingonnat C. Girard N. Chauzy C. Maunoury R. Olivier A. (1993). Hyaluronan and hyaluronectin in the extracellular matrix of human brain tumour stroma. Eur. J. Cancer 29, 1012–1017. 10.1016/S0959-8049, 80214-X 7684596

[B31] Dhandapani M. Gupta S. Mohanty M. Gupta S. K. Dhandapani S. (2016). Trends in cognitive dysfunction following surgery for intracranial tumors. Surg. Neurol. Int. 7, S190–S195. 10.4103/2152-7806.179229 27114854 PMC4825349

[B32] Dwyer C. A. Bi W. L. Viapiano M. S. Matthews R. T. (2014). Brevican knockdown reduces late-stage glioma tumor aggressiveness. J. Neurooncol. 120, 63–72. 10.1007/s11060-014-1541-z 25052349 PMC4177969

[B33] Erickson A. E. Lan Levengood S. K. Sun J. Chang F. C. Zhang M. (2018). Fabrication and characterization of chitosan-hyaluronic acid scaffolds with varying stiffness for glioblastoma cell culture. Adv. Healthc. Mater. 7:e1800295. 10.1002/adhm.201800295 29893067 PMC6116517

[B34] Fangusaro J. (2012). Pediatric high grade glioma: a review and update on tumor clinical characteristics and biology. Front Oncol. 2:105. 10.3389/fonc.2012.00105 22937526 PMC3426754

[B35] Feng X. Xu W. Li Z. Song W. Ding J. Chen X. (2019). Immunomodulatory nanosystems. Adv. Sci. 6:1900101. 10.1002/advs.201900101 31508270 PMC6724480

[B36] Fon D. Zhou K. Ercole F. Fehr F. Marchesan S. Minter M. R.. (2014). Nanofibrous scaffolds releasing a small molecule BDNF-mimetic for the re-direction of endogenous neuroblast migration in the brain. Biomaterials 35, 2692–2712. 10.1016/j.biomaterials.2013.12.016 24406218

[B37] Fourniols T. Randolph L. D. Staub A. Vanvarenberg K. Leprince J. G. Préat V.. (2015). Temozolomide-loaded photopolymerizable PEG-DMA-based hydrogel for the treatment of glioblastoma. J. Control. Release 210, 95–104. 10.1016/j.jconrel.2015.05.272 25982679

[B38] Furnari F. B. Fenton T. Bachoo R. M. Mukasa A. Stommel J. M. Stegh A.. (2007). Malignant astrocytic glioma: genetics, biology, and paths to treatment. Genes Dev. 21, 2683–2710. 10.1101/gad.1596707 17974913

[B39] Furtado D. Björnmalm M. Ayton S. Bush A. I. Kempe K. Caruso F. (2018). Overcoming the blood-brain barrier: the role of nanomaterials in treating neurological diseases. Adv. Mater. Weinheim. 30:e1801362. 10.1002/adma.201801362 30066406

[B40] Ghasemi-Mobarakeh L. Prabhakaran M. P. Morshed M. Nasr-Esfahani M. H. Ramakrishna S. (2008). Electrospun poly(epsilon-caprolactone)/gelatin nanofibrous scaffolds for nerve tissue engineering. Biomaterials 29, 4532–4539. 10.1016/j.biomaterials.2008.08.007 18757094

[B41] Ghuman H. Mauney C. Donnelly J. Massensini A. R. Badylak S. F. Modo M. (2018). Biodegradation of ECM hydrogel promotes endogenous brain tissue restoration in a rat model of stroke. Acta Biomater. 80, 66–84. 10.1016/j.actbio.2018.09.020 30232030 PMC6217851

[B42] Giuliani A. Manescu A. Larsson E. Tromba G. Luongo G. Piattelli A.. (2014). *In vivo* regenerative properties of coralline-derived (biocoral) scaffold grafts in human maxillary defects: demonstrative and comparative study with Beta-tricalcium phosphate and biphasic calcium phosphate by synchrotron radiation x-ray microtomography. Clin. Implant Dent. Relat. Res. 16, 736–750. 10.1111/cid.12039 23350548

[B43] Gordon V. D. Valentine M. T. Gardel M. L. Andor-Ardó D. Dennison S. Bogdanov A. A.. (2003). Measuring the mechanical stress induced by an expanding multicellular tumor system: a case study. Exp. Cell Res. 289, 58–66. 10.1016/s0014-4827(03)00256-8 12941604

[B44] Graham-Gurysh E. Moore K. M. Satterlee A. B. Sheets K. T. Lin F. C. Bachelder E. M.. (2018). Sustained delivery of doxorubicin via acetalated dextran scaffold prevents glioblastoma recurrence after surgical resection. Mol. Pharm. 15, 1309–1318. 10.1021/acs.molpharmaceut.7b01114 29342360 PMC5999333

[B45] Grossman S. A. Reinhard C. Colvin O. M. Chasin M. Brundrett R. Tamargo R. J.. (1992). The intracerebral distribution of BCNU delivered by surgically implanted biodegradable polymers. J. Neurosurg. 76, 640–647. 10.3171/jns.1992.76.4.0640 1545259

[B46] Gulaia V. Kumeiko V. Shved N. Cicinskas E. Rybtsov S. Ruzov A.. (2018). Molecular mechanisms governing the stem cell's fate in brain cancer: factors of stemness and quiescence. Front. Cell Neurosci. 12:388. 10.3389/fncel.2018.00388 30510501 PMC6252330

[B47] Hay E. D. (1993). Extracellular matrix alters epithelial differentiation. Curr. Opin. Cell Biol. 5, 1029–1035. 10.1016/0955-0674, 90088-8 8129940

[B48] Hrapko M. van Dommelen J. A. Peters G. W. Wismans J. S. (2008). The influence of test conditions on characterization of the mechanical properties of brain tissue. J. Biomech. Eng. 130:031003. 10.1115/1.2907746 18532852

[B49] Hu Q. Gao X. Gu G. Kang T. Tu Y. Liu Z.. (2013). Glioma therapy using tumor homing and penetrating peptide-functionalized PEG-PLA nanoparticles loaded with paclitaxel. Biomaterials 34, 5640–5650. 10.1016/j.biomaterials.2013.04.025 23639530

[B50] Huynh N. T. Passirani C. Saulnier P. Benoit J. P. (2009). Lipid nanocapsules: a new platform for nanomedicine. Int. J. Pharm. 379, 201–209. 10.1016/j.ijpharm.2009.04.026 19409468

[B51] Imbeault S. Gauvin L. G. Toeg H. D. Pettit A. Sorbara C. D. Migahed L.. (2009). The extracellular matrix controls gap junction protein expression and function in postnatal hippocampal neural progenitor cells. BMC Neurosci. 10:13. 10.1186/1471-2202-10-13 19236721 PMC2655299

[B52] Isa I. L. Srivastava A. Tiernan D. Owens P. Rooney P. Dockery P.. (2015). Hyaluronic acid based hydrogels attenuate inflammatory receptors and neurotrophins in interleukin-1β induced inflammation model of nucleus pulposus cells. Biomacromolecules 16, 1714–1725. 10.1021/acs.biomac.5b00168 25871410

[B53] Jain A. Kim Y. T. McKeon R. J. Bellamkonda R. V. (2006). In situ gelling hydrogels for conformal repair of spinal cord defects, and local delivery of BDNF after spinal cord injury. Biomaterials 27, 497–504. 10.1016/j.biomaterials.2005.07.008 16099038

[B54] Jain S. Mishra V. Singh P. Dubey P. K. Saraf D. K. Vyas S. P. (2003). RGD-anchored magnetic liposomes for monocytes/neutrophils-mediated brain targeting. Int. J. Pharm. 261, 43–55. 10.1016/s0378-5173(03)00269-2 12878394

[B55] Jaworski S. Sawosz E. Kutwin M. Wierzbicki M. Hinzmann M. Grodzik M.. (2015). *In vitro* and *in vivo* effects of graphene oxide and reduced graphene oxide on glioblastoma. Int. J. Nanomedicine 10, 1585–1596. 10.2147/IJN.S77591 25759581 PMC4346365

[B56] Jia W. Jiang X. Liu W. Wang L. Zhu B. Zhu H.. (2018). Effects of three-dimensional collagen scaffolds on the expression profiles and biological functions of glioma cells. Int. J. Oncol. 52, 1787–1800. 10.3892/ijo.2018.4330 29568859 PMC5919708

[B57] Jiang L. Zhou Q. Mu K. Xie H. Zhu Y. Zhu W.. (2013). pH/temperature sensitive magnetic nanogels conjugated with Cy5.5-labled lactoferrin for MR and fluorescence imaging of glioma in rats. Biomaterials 34, 7418–7428. 10.1016/j.biomaterials.2013.05.078 23810255

[B58] Jin H. Zhao G. Hu J. Ren Q. Yang K. Wan C.. (2017). Melittin-containing hybrid peptide hydrogels for enhanced photothermal therapy of glioblastoma. ACS Appl. Mater. Interfaces 9, 25755–25766. 10.1021/acsami.7b06431 28714303

[B59] Kaufman L. J. Brangwynne C. P. Kasza K. E. Filippidi E. Gordon V. D. Deisboeck T. S.. (2005). Glioma expansion in collagen I matrices: analyzing collagen concentration-dependent growth and motility patterns. Biophys. J. 89, 635–650. 10.1529/biophysj.105.061994 15849239 PMC1366562

[B60] Kearns S. M. Laywell E. D. Kukekov V. K. Steindler D. A. (2003). Extracellular matrix effects on neurosphere cell motility. Exp. Neurol. 182, 240–244. 10.1016/S0014-4886, 00124-9 12821394

[B61] Kerever A. Schnack J. Vellinga D. Ichikawa N. Moon C. Arikawa-Hirasawa E.. (2007). Novel extracellular matrix structures in the neural stem cell niche capture the neurogenic factor fibroblast growth factor 2 from the extracellular milieu. Stem Cells 25, 2146–2157. 10.1634/stemcells.2007-0082 17569787

[B62] Kievit F. M. Cooper A. Jana S. Leung M. C. Wang K. Edmondson D.. (2013). Aligned chitosan-polycaprolactone polyblend nanofibers promote the migration of glioblastoma cells. Adv. Healthc. Mater. 2, 1651–1659. 10.1002/adhm.201300092 23776187 PMC3859701

[B63] Kievit F. M. Florczyk S. J. Leung M. C. Wang K. Wu J. D. Silber J. R.. (2014). Proliferation and enrichment of CD133(+) glioblastoma cancer stem cells on 3D chitosan-alginate scaffolds. Biomaterials 35, 9137–9143. 10.1016/j.biomaterials.2014.07.037 25109438 PMC4167581

[B64] Kim C. F. Jackson E. L. Woolfenden A. E. Lawrence S. Babar I. Vogel S.. (2005). Identification of bronchioalveolar stem cells in normal lung and lung cancer. Cell 121, 823–835. 10.1016/j.cell.2005.03.032 15960971

[B65] Kim K. K. Pack D. W. (2006). Microspheres for drug delivery, in BioMEMS and Biomedical Nanotechnology: Volume I Biological and Biomedical Nanotechnology, eds Ferrari M. Lee A. P. Lee L. J (Boston, MA: Springer US), 19–50.

[B66] Klein E. A. Yin L. Kothapalli D. Castagnino P. Byfield F. J. Xu T.. (2009). Cell-cycle control by physiological matrix elasticity and *in vivo* tissue stiffening. Curr. Biol. 19, 1511–1518. 10.1016/j.cub.2009.07.069 19765988 PMC2755619

[B67] Knott J. C. Mahesparan R. Garcia-Cabrera I. Bølge Tysnes B. Edvardsen K. Ness G. O.. (1998). Stimulation of extracellular matrix components in the normal brain by invading glioma cells. Int. J. Cancer 75, 864–872. 9506531 10.1002/(sici)1097-0215(19980316)75:6<864::aid-ijc8>3.0.co;2-t

[B68] Kondo T. Setoguchi T. Taga T. (2004). Persistence of a small subpopulation of cancer stem-like cells in the C6 glioma cell line. Proc. Natl. Acad. Sci. U.S.A. 101, 781–786. 10.1073/pnas.0307618100 14711994 PMC321758

[B69] Konishi Y. Muragaki Y. Iseki H. Mitsuhashi N. Okada Y. (2012). Patterns of intracranial glioblastoma recurrence after aggressive surgical resection and adjuvant management: retrospective analysis of 43 cases. Neurol. Med. Chir. (Tokyo) 52, 577–586. 10.2176/nmc.52.577 22976141

[B70] Koochekpour S. Pilkington G. J. Merzak A. (1995). Hyaluronic acid/CD44H interaction induces cell detachment and stimulates migration and invasion of human glioma cells *in vitro*. Int. J. Cancer 63, 450–454. 10.1002/ijc.2910630325 7591247

[B71] Kunert P. Smolarek B. Marchel A. (2011). Facial nerve damage following surgery for cerebellopontine angle tumours. Prevention and comprehensive treatment. Neurol. Neurochir. Pol. 45, 480–488. 10.1016/S0028-3843, 60317-0 22127944

[B72] Lathia J. D. Li M. Hall P. E. Gallagher J. Hale J. S. Wu Q.. (2012). Laminin alpha 2 enables glioblastoma stem cell growth. Ann. Neurol. 72, 766–778. 10.1002/ana.23674 23280793 PMC3615417

[B73] Lathia J. D. Patton B. Eckley D. M. Magnus T. Mughal M. R. Sasaki T.. (2007). Patterns of laminins and integrins in the embryonic ventricular zone of the CNS. J. Comp. Neurol. 505, 630–643. 10.1002/cne.21520 17948866

[B74] Lau L. W. Cua R. Keough M. B. Haylock-Jacobs S. Yong V. W. (2013). Pathophysiology of the brain extracellular matrix: a new target for remyelination. Nat. Rev. Neurosci. 14, 722–729. 10.1038/nrn3550 23985834

[B75] Lee E. J. Kasper F. K. Mikos A. G. (2014). Biomaterials for tissue engineering. Ann. Biomed. Eng. 42, 323–337. 10.1007/s10439-013-0859-6 23820768 PMC3844045

[B76] Lee E. X. Lam D. H. Wu C. Yang J. Tham C. K. Ng W. H.. (2011). Glioma gene therapy using induced pluripotent stem cell derived neural stem cells. Mol. Pharm. 8, 1515–1524. 10.1021/mp200127u 21755959

[B77] Lee K. Y. Mooney D. J. (2012). Alginate: properties and biomedical applications. Prog. Polym. Sci. 37, 106–126. 10.1016/j.progpolymsci.2011.06.003 22125349 PMC3223967

[B78] Liu D. Pearlman E. Diaconu E. Guo K. Mori H. Haqqi T.. (1996). Expression of hyaluronidase by tumor cells induces angiogenesis *in vivo*. Proc. Natl. Acad. Sci. U.S.A. 93, 7832–7837. 10.1073/pnas.93.15.7832 8755562 PMC38834

[B79] Mahmoudi K. Bouras A. Bozec D. Ivkov R. Hadjipanayis C. (2018). Magnetic hyperthermia therapy for the treatment of glioblastoma: a review of the therapy's history, efficacy and application in humans. Int. J. Hyperthermia 34, 1316–1328. 10.1080/02656736.2018.1430867 29353516 PMC6078833

[B80] Maier-Hauff K. Rothe R. Scholz R. Gneveckow U. Wust P. Thiesen B.. (2007). Intracranial thermotherapy using magnetic nanoparticles combined with external beam radiotherapy: results of a feasibility study on patients with glioblastoma multiforme. J. Neurooncol. 81, 53–60. 10.1007/s11060-006-9195-0 16773216

[B81] Malinovskaya Y. Melnikov P. Baklaushev V. Gabashvili A. Osipova N. Mantrov S.. (2017). Delivery of doxorubicin-loaded PLGA nanoparticles into U87 human glioblastoma cells. Int. J. Pharm. 524, 77–90. 10.1016/j.ijpharm.2017.03.049 28359811

[B82] Mallick S. Benson R. Hakim A. Rath G. K. (2016). Management of glioblastoma after recurrence: a changing paradigm. J. Egypt. Natl. Canc. Inst. 28, 199–210. 10.1016/j.jnci.2016.07.001 27476474

[B83] Martínez-Ramos C. Vallés-Lluch A. Verdugo J. M. Ribelles J. L. Barcia Albacar J. A. Orts A. B.. (2012). Channeled scaffolds implanted in adult rat brain. J. Biomed. Mater. Res. A 100, 3276–3286. 10.1002/jbm.a.34273 22733596

[B84] Mecham R. P. (2012). Overview of extracellular matrix. Curr. Protoc. Cell Biol. Chapter 10:11 10.1002/0471143030.cb1001s57 18228295

[B85] Mercier F. (2016). Fractones: extracellular matrix niche controlling stem cell fate and growth factor activity in the brain in health and disease. Cell. Mol. Life Sci. 73, 4661–4674. 10.1007/s00018-016-2314-y 27475964 PMC11108427

[B86] Mikhailova V. Gulaia V. Tiasto V. Rybtsov S. Yatsunskaya M. Kagansky A. (2018). Towards an advanced cell-based *in vitro* glioma model system. AIMS Genet. 5, 91–112. 10.3934/genet.2018.2.91 31435515 PMC6698577

[B87] Miroshnikova Y. A. Mouw J. K. Barnes J. M. Pickup M. W. Lakins J. N. Kim Y.. (2016). Tissue mechanics promote IDH1-dependent HIF1alpha-tenascin C feedback to regulate glioblastoma aggression. Nat. Cell Biol. 18, 1336–1345. 10.1038/ncb3429 27820599 PMC5361403

[B88] Morshed R. A. Cheng Y. Auffinger B. Wegscheid M. L. Lesniak M. S. (2013). The potential of polymeric micelles in the context of glioblastoma therapy. Front. Pharmacol. 4:157. 10.3389/fphar.2013.00157 24416018 PMC3874582

[B89] Mouw J. K. Ou G. Weaver V. M. (2014). Extracellular matrix assembly: a multiscale deconstruction. Nat. Rev. Mol. Cell Biol. 15, 771–785. 10.1038/nrm3902 25370693 PMC4682873

[B90] Müller R. H. Radtke M. Wissing S. A. (2002). Solid lipid nanoparticles (SLN) and nanostructured lipid carriers (NLC) in cosmetic and dermatological preparations. Adv. Drug Deliv. Rev. 54, S131–S155. 10.1016/S0169-409X(02)00118-7 12460720

[B91] Nakamizo A. Marini F. Amano T. Khan A. Studeny M. Gumin J.. (2005). Human bone marrow-derived mesenchymal stem cells in the treatment of gliomas. Cancer Res. 65, 3307–3318. 10.1158/0008-5472.CAN-04-1874 15833864

[B92] Nam L. Coll C. Erthal L. C. S. de la Torre C. Serrano D. Martínez-Máñez R.. (2018). Drug delivery nanosystems for the localized treatment of glioblastoma multiforme. Materials (Basel). 11:e779. 10.3390/ma11050779 29751640 PMC5978156

[B93] Ng J. C. H. See A. A. Q. Ang T. Y. Tan L. Y. R. Ang B. T. King N. K. K. (2019). Effects of surgery on neurocognitive function in patients with glioma: a meta-analysis of immediate post-operative and long-term follow-up neurocognitive outcomes. J. Neurooncol. 141, 167–182. 10.1007/s11060-018-03023-9 30446902

[B94] Nicholson C. Syková E. (1998). Extracellular space structure revealed by diffusion analysis. Trends Neurosci. 21, 207–215. 10.1016/S0166-223601261-2 9610885

[B95] Nih L. R. Gojgini S. Carmichael S. T. Segura T. (2018). Dual-function injectable angiogenic biomaterial for the repair of brain tissue following stroke. Nat. Mater. 17, 642–651. 10.1038/s41563-018-0083-8 29784996 PMC6019573

[B96] Novak U. Kaye A. H. (2000). Extracellular matrix and the brain: components and function. J. Clin. Neurosci. 7, 280–290. 10.1054/jocn.1999.0212 10938601

[B97] Novak U. Stylli S. S. Kaye A. H. Lepperdinger G. (1999). Hyaluronidase-2 overexpression accelerates intracerebral but not subcutaneous tumor formation of murine astrocytoma cells. Cancer Res. 59, 6246–6250. 10626819

[B98] Nyagilo J. Piccirillo S. Sun Y. Bui L. Regmi N. Wright J. L. (2017). Abstract A54: extracellular matrix geometry and 3D spatial confinement trigger diverse mechanisms of primary human glioblastoma cell migration. Cancer Res. 77:A54 10.1158/1538-7445.EPSO16-A54

[B99] Nyga A. Cheema U. Loizidou M. (2011). 3D tumour models: novel *in vitro* approaches to cancer studies. J. Cell Commun. Signal 5, 239–248. 10.1007/s12079-011-0132-4 21499821 PMC3145874

[B100] Ostrom Q. T. Bauchet L. Davis F. G. Deltour I. Fisher J. L. Langer C. E.. (2014). The epidemiology of glioma in adults: a “state of the science” review. Neuro-oncol. 16, 896–913. 10.1093/neuonc/nou087 24842956 PMC4057143

[B101] Ozeki T. Kaneko D. Hashizawa K. Imai Y. Tagami T. Okada H. (2012). Combination therapy of surgical tumor resection with implantation of a hydrogel containing camptothecin-loaded poly(lactic-co-glycolic acid) microspheres in a C6 rat glioma model. Biol. Pharm. Bull. 35, 545–550. 10.1248/bpb.35.545 22466559

[B102] Paul C. D. Mistriotis P. Konstantopoulos K. (2017). Cancer cell motility: lessons from migration in confined spaces. Nat. Rev. Cancer 17, 131–140. 10.1038/nrc.2016.123 27909339 PMC5364498

[B103] Pawar K. Prang P. Müller R. Caioni M. Bogdahn U. Kunz W.. (2015). Intrinsic and extrinsic determinants of central nervous system axon outgrowth into alginate-based anisotropic hydrogels. Acta Biomater. 27, 131–139. 10.1016/j.actbio.2015.08.032 26310676

[B104] Pedron S. Becka E. Harley B. A. (2013). Regulation of glioma cell phenotype in 3D matrices by hyaluronic acid. Biomaterials 34, 7408–7417. 10.1016/j.biomaterials.2013.06.024 23827186

[B105] Pedron S. Hanselman J. S. Schroeder M. A. Sarkaria J. N. Harley B. A. C. (2017). Extracellular hyaluronic acid influences the efficacy of EGFR tyrosine kinase inhibitors in a biomaterial model of glioblastoma. Adv. Healthc. Mater. 6:1700529 10.1002/adhm.201700529 28766870 PMC5726872

[B106] Pinel S. Thomas N. Boura C. Barberi-Heyob M. (2019). Approaches to physical stimulation of metallic nanoparticles for glioblastoma treatment. Adv. Drug Deliv. Rev. 138, 344–357. 10.1016/j.addr.2018.10.013 30414495

[B107] Pinto L. W. Araújo M. B. Vettore A. L. Wernersbach L. Leite A. C. Chimelli L. M.. (2008). Glioblastomas: correlation between oligodendroglial components, genetic abnormalities, and prognosis. Virchows Arch. 452, 481–490. 10.1007/s00428-007-0562-9 18351387

[B108] Plopper G. (2015). The extracellular matrix and cell adhesion, in Cells, eds Plopper G. Sikorski E (Burlington, MA: Jones and Bartlett), 831–889.

[B109] Pogoda K. Chin L. Georges P. C. Byfield F. J. Bucki R. Kim R.. (2014). Compression stiffening of brain and its effect on mechanosensing by glioma cells. New J. Phys. 16:075002. 10.1088/1367-2630/16/7/075002 25844043 PMC4380293

[B110] Puente P. Fettig N. Luderer M. J. Jin A. Shah S. Muz B.. (2018). Injectable hydrogels for localized chemotherapy and radiotherapy in brain tumors. J. Pharm. Sci. 107, 922–933. 10.1016/j.xphs.2017.10.042 29162424 PMC6093750

[B111] Pulgar V. M. (2018). Transcytosis to cross the blood brain barrier, new advancements and challenges. Front. Neurosci. 12:1019. 10.3389/fnins.2018.01019 30686985 PMC6337067

[B112] Qi N. Cai C. Zhang W. Niu Y. Yang J. Wang L.. (2014). Sustained delivery of cytarabine-loaded vesicular phospholipid gels for treatment of xenografted glioma. Int. J. Pharm. 472, 48–55. 10.1016/j.ijpharm.2014.06.005 24914829

[B113] Qi N. Tang X. Lin X. Gu P. Cai C. Xu H.. (2012). Sterilization stability of vesicular phospholipid gels loaded with cytarabine for brain implant. Int. J. Pharm. 427, 234–241. 10.1016/j.ijpharm.2012.02.008 22349049

[B114] Ramachandran R. Junnuthula V. R. Gowd G. S. Ashokan A. Thomas J. Peethambaran R.. (2017). Theranostic 3-Dimensional nano brain-implant for prolonged and localized treatment of recurrent glioma. Sci. Rep. 7:43271. 10.1038/srep43271 28262735 PMC5338016

[B115] Rauch U. (2004). Extracellular matrix components associated with remodeling processes in brain. Cell. Mol. Life Sci. 61, 2031–2045. 10.1007/s00018-004-4043-x 15316653 PMC11138714

[B116] Read T. A. Sorensen D. R. Mahesparan R. Enger P. O. Timpl R. Olsen B. R.. (2001). Local endostatin treatment of gliomas administered by microencapsulated producer cells. Nat. Biotechnol. 19, 29–34. 10.1038/83471 11135548

[B117] Reinhard J. Brösicke N. Theocharidis U. Faissner A. (2016). The extracellular matrix niche microenvironment of neural and cancer stem cells in the brain. Int. J. Biochem. Cell Biol. 81, 174–183. 10.1016/j.biocel.2016.05.002 27157088

[B118] Rowland M. J. Parkins C. C. McAbee J. H. Kolb A. K. Hein R. Loh X. J.. (2018). An adherent tissue-inspired hydrogel delivery vehicle utilised in primary human glioma models. Biomaterials 179, 199–208. 10.1016/j.biomaterials.2018.05.054 30037456

[B119] Saito K. Yamasaki K. Yokogami K. Ivanova A. Takeishi G. Sato Y.. (2017). Eosinophilic meningitis triggered by implanted Gliadel wafers: case report. J. Neurosurg. 126, 1783–1787. 10.3171/2016.4.JNS152771 27285546

[B120] Sameshima T. Nabeshima K. Toole B. P. Yokogami K. Okada Y. Goya T.. (2000). Glioma cell extracellular matrix metalloproteinase inducer (EMMPRIN) (CD147) stimulates production of membrane-type matrix metalloproteinases and activated gelatinase A in co-cultures with brain-derived fibroblasts. Cancer Lett. 157, 177–184. 10.1016/S0304-3835, 00485-7 10936678

[B121] Scanga V. I. Goraltchouk A. Nussaiba N. Shoichet M. S. Morshead C. M. (2010). Biomaterials for neural-tissue engineering—Chitosan supports the survival, migration, and differentiation of adult-derived neural stem and progenitor cells. Can. J. Chem. 88, 277–287. 10.1139/v09-171

[B122] Seidenbecher C. I. Richter K. Rauch U. Fässler R. Garner C. C. Gundelfinger E. D. (1995). Brevican, a chondroitin sulfate proteoglycan of rat brain, occurs as secreted and cell surface glycosylphosphatidylinositol-anchored isoforms. J. Biol. Chem. 270, 27206–27212. 10.1074/jbc.270.45.27206 7592978

[B123] Senkov O. Andjus P. Radenovic L. Soriano E. Dityatev A. (2014). Neural ECM molecules in synaptic plasticity, learning, and memory. Prog. Brain Res. 214, 53–80. 10.1016/B978-0-444-63486-3.00003-7 25410353

[B124] Sim H. Hu B. Viapiano M. S. (2009). Reduced expression of the hyaluronan and proteoglycan link proteins in malignant gliomas. J. Biol. Chem. 284, 26547–26556. 10.1074/jbc.M109.013185 19633295 PMC2785343

[B125] Später T. Frueh F. S. Metzger W. Menger M. D. Laschke M. W. (2018). *In vivo* biocompatibility, vascularization, and incorporation of Integra((R)) dermal regenerative template and flowable wound matrix. J. Biomed. Mater. Res. B Appl. Biomater. 106, 52–60. 10.1002/jbm.b.33813 27862914

[B126] Srivastava A. Yadav T. Sharma S. Nayak A. Kumari A. Mishra N. (2016). Polymers in drug delivery. J. Biosci. Med. 4, 69–84. 10.4236/jbm.2016.41009

[B127] Stephan M. T. Irvine D. J. (2011). Enhancing cell therapies from the outside in: cell surface engineering using synthetic nanomaterials. Nano Today 6, 309–325. 10.1016/j.nantod.2011.04.001 21826117 PMC3148657

[B128] Stoppel W. L. Ghezzi C. E. McNamara S. L. Black L. D. III. Kaplan D. L. (2015). Clinical applications of naturally derived biopolymer-based scaffolds for regenerative medicine. Ann. Biomed. Eng. 43, 657–680. 10.1007/s10439-014-1206-2 25537688 PMC8196399

[B129] Sturm D. Pfister S. M. Jones D. T. W. (2017). Pediatric gliomas: current concepts on diagnosis, biology, and clinical management. J. Clin. Oncol. 35, 2370–2377. 10.1200/JCO.2017.73.0242 28640698

[B130] Sun Y. Pollard S. Conti L. Toselli M. Biella G. Parkin G.. (2008). Long-term tripotent differentiation capacity of human neural stem (NS) cells in adherent culture. Mol. Cell Neurosci. 38, 245–258. 10.1016/j.mcn.2008.02.014 18450476

[B131] Suri V. Das P. Pathak P. Jain A. Sharma M. C. Borkar S. A.. (2009). Pediatric glioblastomas: a histopathological and molecular genetic study. Neurooncology 11, 274–280. 10.1215/15228517-2008-092 18981259 PMC2718971

[B132] Theocharis A. D. Skandalis S. S. Tzanakakis G. N. Karamanos N. K. (2010). Proteoglycans in health and disease: novel roles for proteoglycans in malignancy and their pharmacological targeting. FEBS J. 277, 3904–3923. 10.1111/j.1742-4658.2010.07800.x 20840587

[B133] Tian W. Ying X. Du J. Guo J. Men Y. Zhang Y.. (2010). Enhanced efficacy of functionalized epirubicin liposomes in treating brain glioma-bearing rats. Eur. J. Pharm. Sci. 41, 232–243. 10.1016/j.ejps.2010.06.008 20600880

[B134] Trotter J. Karram K. Nishiyama A. (2010). NG2 cells: properties, progeny and origin. Brain Res. Rev. 63, 72–82. 10.1016/j.brainresrev.2009.12.006 20043946 PMC2862831

[B135] Tseng Y. Y. Kao Y. C. Liao J. Y. Chen W. A. Liu S. J. (2013). Biodegradable drug-eluting poly[lactic-co-glycol acid] nanofibers for the sustainable delivery of vancomycin to brain tissue: *in vitro* and *in vivo* studies. ACS Chem. Neurosci. 4, 1314–1321. 10.1021/cn400108q 23815098 PMC3778423

[B136] Turley E. A. Hossain M. Z. Sorokan T. Jordan L. M. Nagy J. I. (1994). Astrocyte and microglial motility *in vitro* is functionally dependent on the hyaluronan receptor RHAMM. Glia 12, 68–80. 10.1002/glia.440120109 7531178

[B137] Tyler B. Fowers K. D. Li K. W. Recinos V. R. Caplan J. M. Hdeib A.. (2010). A thermal gel depot for local delivery of paclitaxel to treat experimental brain tumors in rats. J. Neurosurg. 113, 210–217. 10.3171/2009.11.JNS08162 20001591

[B138] Ulrich T. A. de Juan Pardo E. M. Kumar S. (2009). The mechanical rigidity of the extracellular matrix regulates the structure, motility, and proliferation of glioma cells. Cancer Res. 69, 4167–4174. 10.1158/0008-5472.CAN-08-4859 19435897 PMC2727355

[B139] Varga I. Hutóczki G. Szemcsák C. D. Zahuczky G. Tóth J. Adamecz Z.. (2012). Brevican, neurocan, tenascin-C and versican are mainly responsible for the invasiveness of low-grade astrocytoma. Pathol. Oncol. Res. 18, 413–420. 10.1007/s12253-011-9461-0 21997179

[B140] Veretennikoff K. Walker D. Biggs V. Robinson G. (2017). Changes in cognition and decision making capacity following brain tumour resection: illustrated with two cases. Brain Sci. 7:E122. 10.3390/brainsci7100122 28946652 PMC5664049

[B141] Vinchon-Petit S. Jarnet D. Michalak S. Lewis A. Benoit J. P. Menei P. (2010). Local implantation of doxorubicin drug eluting beads in rat glioma. Int. J. Pharm. 402, 184–189. 10.1016/j.ijpharm.2010.09.013 20863875

[B142] Wang C. Tong X. Jiang X. Yang F. (2017). Effect of matrix metalloproteinase-mediated matrix degradation on glioblastoma cell behavior in 3D PEG-based hydrogels. J. Biomed. Mater. Res. A 105, 770–778. 10.1002/jbm.a.35947 27770562 PMC5276739

[B143] Wang G. Wang J. J. Tang X. J. Du L. Li F. (2016). *In vitro* and *in vivo* evaluation of functionalized chitosan-Pluronic micelles loaded with myricetin on glioblastoma cancer. Nanomedicine 12, 1263–1278. 10.1016/j.nano.2016.02.004 26970027

[B144] Wang Y. Chang N. Zhang T. Liu H. Ma W. Chu Q.. (2010). Overexpression of human CAP10-like protein 46 KD in T-acute lymphoblastic leukemia and acute myelogenous leukemia. Genet. Test. Mol. Biomarkers 14, 127–133. 10.1089/gtmb.2009.0145 20143914

[B145] Watanabe A. Mabuchi T. Satoh E. Furuya K. Zhang L. Maeda S.. (2006). Expression of syndecans, a heparan sulfate proteoglycan, in malignant gliomas: participation of nuclear factor-kappaB in upregulation of syndecan-1 expression. J. Neurooncol. 77, 25–32. 10.1007/s11060-005-9010-3 16132527

[B146] Wiranowska M. Ladd S. Smith S. R. Gottschall P. E. (2006). CD44 adhesion molecule and neuro-glial proteoglycan NG2 as invasive markers of glioma. Brain Cell Biol. 35, 159–172. 10.1007/s11068-007-9009-0 17957481

[B147] Wu M. P. Tamada J. A. Brem H. Langer R. (1994). *In vivo* versus *in vitro* degradation of controlled release polymers for intracranial surgical therapy. J. Biomed. Mater. Res. 28, 387–395. 10.1002/jbm.820280314 8077254

[B148] Xiao H. Yan L. Dempsey E. M. Song W. Qi R. Li W. (2018). Recent progress in polymer-based platinum drug delivery systems. Prog. Polym. Sci. 87, 70–106. 10.1016/j.progpolymsci.2018.07.004

[B149] Xie H. Zhu Y. Jiang W. Zhou Q. Yang H. Gu N.. (2011). Lactoferrin-conjugated superparamagnetic iron oxide nanoparticles as a specific MRI contrast agent for detection of brain glioma *in vivo*. Biomaterials 32, 495–502. 10.1016/j.biomaterials.2010.09.024 20970851

[B150] Xie Y. T. Du Y. Z. Yuan H. Hu F. Q. (2012). Brain-targeting study of stearic acid-grafted chitosan micelle drug-delivery system. Int. J. Nanomed. 7, 3235–3244. 10.2147/IJN.S32701 22802685 PMC3396390

[B151] Xing W. K. Shao C. Qi Z. Y. Yang C. Wang Z. (2015). The role of Gliadel wafers in the treatment of newly diagnosed GBM: a meta-analysis. Drug Des. Devel. Ther. 9, 3341–3348. 10.2147/DDDT.S85943 26170620 PMC4492653

[B152] Xiong A. Kundu S. Forsberg-Nilsson K. (2014). Heparan sulfate in the regulation of neural differentiation and glioma development. FEBS J. 281, 4993–5008. 10.1111/febs.13097 25284049

[B153] Yamaguchi Y. (2000). Lecticans: organizers of the brain extracellular matrix. Cell Mol. Life Sci. 57, 276–289. 10.1007/PL00000690 10766023 PMC11146776

[B154] Yamahara T. Numa Y. Oishi T. Kawaguchi T. Seno T. Asai A.. (2010). Morphological and flow cytometric analysis of cell infiltration in glioblastoma: a comparison of autopsy brain and neuroimaging. Brain Tumor Pathol. 27, 81–87. 10.1007/s10014-010-0275-7 21046309

[B155] Yang G. Murashige D. S. Humphrey S. J. James D. E. (2015). A positive feedback loop between Akt and mTORC2 via SIN1 phosphorylation. Cell Rep. 12, 937–943. 10.1016/j.celrep.2015.07.016 26235620

[B156] Yang J. Li Y. Zhang T. Zhang X. (2016). Development of bioactive materials for glioblastoma therapy. Bioact Mater. 1, 29–38. 10.1016/j.bioactmat.2016.03.003 29744393 PMC5883963

[B157] Yu C. Griffiths L. R. Haupt L. M. (2017). Exploiting heparan sulfate proteoglycans in human neurogenesis-controlling lineage specification and fate. Front Integr. Neurosci. 11:28. 10.3389/fnint.2017.00028 29089873 PMC5650988

[B158] Yu Q. Stamenkovic I. (1999). Localization of matrix metalloproteinase 9 to the cell surface provides a mechanism for CD44-mediated tumor invasion. Genes Dev. 13, 35–48. 10.1101/gad.13.1.35 9887098 PMC316376

[B159] Yu Y. Xu Q. He S. Xiong H. Zhang Q. Xu W. (2019). Recent advances in delivery of photosensitive metal-based drugs. Coord. Chem. Rev. 387, 154–179. 10.1016/j.ccr.2019.01.020

[B160] Zamecnik J. (2005). The extracellular space and matrix of gliomas. Acta Neuropathol. 110, 435–442. 10.1007/s00401-005-1078-5 16175354

[B161] Zhao M. Danhier F. Bastiancich C. Joudiou N. Ganipineni L. P. Tsakiris N.. (2018). Post-resection treatment of glioblastoma with an injectable nanomedicine-loaded photopolymerizable hydrogel induces long-term survival. Int. J. Pharm. 548, 522–529. 10.1016/j.ijpharm.2018.07.033 30017818

